# Whole genome analysis of p38 SAPK-mediated gene expression upon stress

**DOI:** 10.1186/1471-2164-11-144

**Published:** 2010-03-01

**Authors:** Isabel Ferreiro, Manel Joaquin, Abul Islam, Gonzalo Gomez-Lopez, Montserrat Barragan, Luís Lombardía, Orlando Domínguez, David G Pisano, Nuria Lopez-Bigas, Angel R Nebreda, Francesc Posas

**Affiliations:** 1 Cell Signaling Unit, Universitat Pompeu Fabra (UPF) Dr aiguader 88, Barcelona 08003, Spain; 2 Research Unit on Biomedical Informatics. Departament de Ciències Experimentals i de la Salut (DCEXS), Universitat Pompeu Fabra (UPF) Dr aiguader 88, Barcelona 08003, Spain; 3 Bioinformatics Unit, Centro Nacional de Investigaciones Oncológicas (CNIO), Melchor Fernández Almagro 3, Madrid 28029, Spain; 4 Molecular Diagnostics Unit, Centro Nacional de Investigaciones Oncológicas (CNIO), Melchor Fernández Almagro 3, Madrid 28029, Spain; 5 Genomics Unit, Centro Nacional de Investigaciones Oncológicas (CNIO), Melchor Fernández Almagro 3, Madrid 28029, Spain; 6 Signalling and Cell Cycle Group, Centro Nacional de Investigaciones Oncológicas (CNIO), Melchor Fernández Almagro 3, Madrid 28029, Spain

## Abstract

**Background:**

Cells have the ability to respond and adapt to environmental changes through activation of stress-activated protein kinases (SAPKs). Although p38 SAPK signalling is known to participate in the regulation of gene expression little is known on the molecular mechanisms used by this SAPK to regulate stress-responsive genes and the overall set of genes regulated by p38 in response to different stimuli.

**Results:**

Here, we report a whole genome expression analyses on mouse embryonic fibroblasts (MEFs) treated with three different p38 SAPK activating-stimuli, namely osmostress, the cytokine TNFα and the protein synthesis inhibitor anisomycin. We have found that the activation kinetics of p38α SAPK in response to these insults is different and also leads to a complex gene pattern response specific for a given stress with a restricted set of overlapping genes. In addition, we have analysed the contribution of p38α the major p38 family member present in MEFs, to the overall stress-induced transcriptional response by using both a chemical inhibitor (SB203580) and p38α deficient (p38α^-/-^) MEFs. We show here that p38 SAPK dependency ranged between 60% and 88% depending on the treatments and that there is a very good overlap between the inhibitor treatment and the ko cells. Furthermore, we have found that the dependency of SAPK varies depending on the time the cells are subjected to osmostress.

**Conclusions:**

Our genome-wide transcriptional analyses shows a selective response to specific stimuli and a restricted common response of up to 20% of the stress up-regulated early genes that involves an important set of transcription factors, which might be critical for either cell adaptation or preparation for continuous extra-cellular changes. Interestingly, up to 85% of the up-regulated genes are under the transcriptional control of p38 SAPK. Thus, activation of p38 SAPK is critical to elicit the early gene expression program required for cell adaptation to stress.

## Background

Cells have the ability to respond and adapt to environmental changes through the activation of stress-activated protein kinases (SAPKs). A well-studied prototype of SAPK is the budding yeast *Saccharomyces cerevisae *Hog1. Upon osmotic shock, two complex molecular osmosensing systems located at the plasma membrane convert the extracellular information into a signal that leads to a rapid and transient Hog1 activation and nuclear translocation of this SAPK [1]. The activity of Hog1 is essential for adaptation to osmostress and regulates key biological processes such us cell cycle and gene expression [2]. In response to osmostress, the Hog1 SAPK is a key regulatory element for the activation of a specific osmostress-induced gene program. Genome-wide transcription studies have revealed that close to a 7% of the whole budding yeast genome had significant and transient changes in the expression levels of the genes after osmotic shock. Moreover, up to 70% of those regulated genes depend on the Hog1 SAPK activity. Taken together, the data in yeast indicate that there is a key role for SAPKs in reprogramming the gene expression capacity of cells in response to external stimulus [3,4]. The mammalian structural and functional homolog of the Hog1 SAPK is the p38 family of SAPKs. It is worth noting that heterologous expression of the p38 SAPK is able to rescue the sensitivity to osmostress of a *hog1 *deficient yeast strain [5]. In contrast to Hog1, which is activated primarily upon osmostress, mammalian p38 SAPKs are activated in response to many insults such as infection, inflammatory cytokines, anisomycin and by a broad range of environmental stresses (e.g., osmostress, UV, heat stress, heavy metals, etc). Four genes encode p38 SAPKs in mammals. However, whereas p38α and p38β seem to have overlapping functions and are widely expressed, being p38α the most abundant protein in all tissues, p38γ and p38δ are expressed in specific cell types and are likely to have specialised functions. Moreover p38 SAPKs have been involved in several biological processes such as inflammation, cell growth, cell differentiation, cell cycle and cell death [6-8]. Although it has been shown that p38 MAPK signalling participates in the regulation of gene transcription little is known on the molecular mechanisms used by this SAPK to regulate stress-responsive gene expression as well as the overall set of genes regulated by p38 in response to different stimuli [9]. p38 SAPK transcriptional profiles have been described in primary endothelial cells from human umbilical veins and rat fibroblasts-like synuviocytes after long term incubation with TNFα [10,11], in response to the inhibition of the p38 SAPK in primary human keratinocytes [12] and proliferating cardiomyocites [13]. However comprehensive genome-wide transcription studies describing the involvement of the p38 SAPK on immediate stress-induced genes or a comparative analysis of the genes that respond to different stimuli under the SAPK activation have not been reported to date.

To gain a deeper knowledge on the p38 SAPK transcriptome we have performed whole genome microarray analysis using mouse embryonic fibroblasts (MEFs) treated with different p38 SAPK activators, namely osmostress, the cytokine TNFα and the protein synthesis inhibitor anisomycin. In addition, we have analysed the contribution of p38α, the major p38 family member present in MEFs to the overall transcription in response to those stimuli by using both the chemical inhibitor SB203580 and p38α knockout MEFs. The results obtained show a complex gene pattern response specific for a given stress with a restricted set of overlapping genes being both highly dependent on p38 SAPK activity.

## Results

### The activation kinetics of p38α SAPK in response to external insults depends on the stimuli

**Figure 1:**
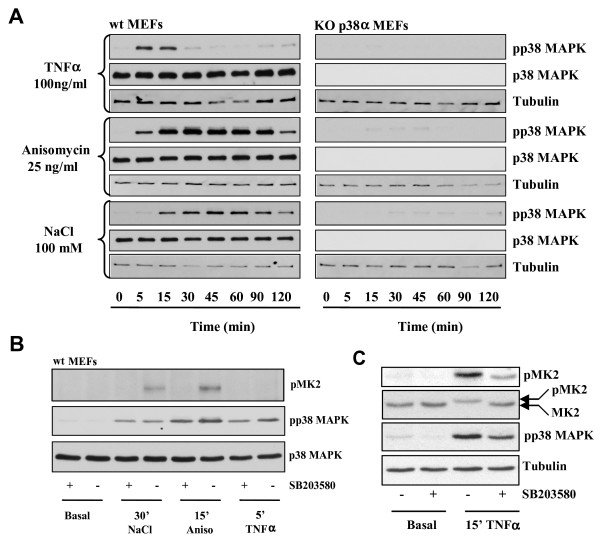
**p38 SAPK activation in wt MEFs and p38α^-/- ^knock out MEFs**. A. wt and p38α^-/- ^MEFs cells were stimulated with 100 ng/ml TNFα, 25 ng/ml anisomycin and 100 mM NaCl for the indicated times. Cleared cell lysates were subsequently analysed by western blotting with antibodies that recognise the phosphorylated and active form of p38 SAPK, total p38α SAPK or tubulin. Representative western blots are shown. B. wt MEFs were pre-treated or not with 10 μM SB203502 prior to the addition of the p38 SAPK activators for the indicated times. Cleared cell lysates were subsequently analysed by western blotting with antibodies that recognise phosphorylated MK2, the phosphorylated and active form of p38 SAPK and total p38α SAPK. Representative western blots are shown. C. As in B western blots were analysed with antibodies raised against total and phosphorylated MK2, pp38 SAPK and tubulin. Rrepresentatives western blots are shown.

To analyse the kinetics of activation of p38 SAPK in response to several stimuli, we assessed p38 SAPK phosphorylation on the activating residues Thr180 and Tyr182 by western blot using a phospho-specific antibody. Addition of TNFα (100 ng/ml) to MEFs induced a rapid and sharp activation of the SAPK followed by a quick down-regulation. On the other hand, anisomycin, at a concentration that does not fully block protein synthesis (25 ng/ml) [14], was able to activate p38 SAPK to a greater extent, peaking at 30 minutes, decreasing slowly afterwards and with a clear reduction after 120 minutes. An hyperosmotic shock induced by NaCl (100 mM) was able to activate p38 SAPK with a slower kinetics than TNFα with a peak at 45 minutes followed by a down-regulation. Anisomycin had a similar kinetic to that seen for osmostress. Moreover, both anisomycin and NaCl induced longer p38 SAPK activation than TNFα over time (Figure 1A, left panels). Therefore, all the stimuli tested were able to transiently activate p38 SAPK although with different kinetics.

Importantly, the three stimuli did not induce detectable p38 SAPK activation in p38α^-/- ^MEFs, indicating that these cells represent a useful tool to study the loss of function of p38 SAPK (Figure 1A, right panels). We also confirmed that the chemical inhibitor SB203580 did no prevent p38α phosphorylation but impaired the phosphorylation of the p38 SAPK substrate MK2, indicating that was able to inhibit p38 activation (Figure 1B). We were not eble to detect MK2 phosphorylation upon TNFα addition at 5'. Therefore we extended the incubation time with this cytokine. After 15' of TNFα addition we could detect a slow migrating form of the MK2 protein that correlated with its phosphorylation. Both events were prevented by the p38 SAPK chemical inhibitor SB203580 (Figure 1C).

### Activation of p38 by different stimuli leads to the regulation of different sets of genes

**Figure 2:**
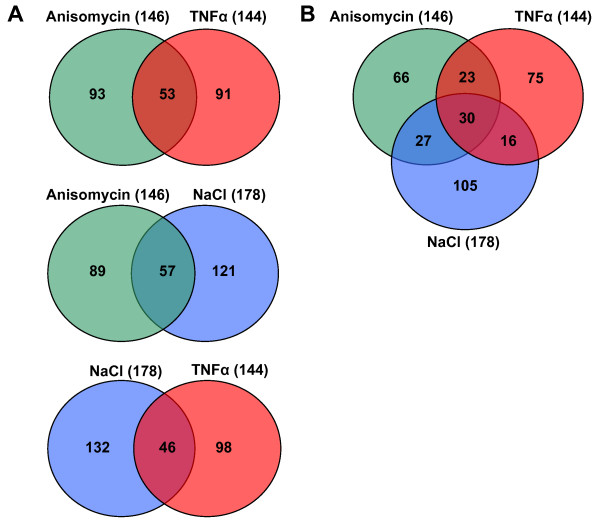
**Genes up-regulated by anisomycin, TNFα and NaCl**. A. Venn diagrams show the total numbers of genes up-regulated more than 2.5 fold and the overlapping between the treatments. B. Venn diagram showing the gene overlapping among the three treatments used.

To analyse the gene expression changes mediated by p38 SAPK in response to different stimuli, wild type and p38α knockout MEFs were treated with TNFα (100 ng/ml) for 45 minutes, anisomycin (25 ng/ml) for 45 minutes and NaCl (100 mM) for 2 h. In addition, wild type MEFs were also pre-treated with 10 μM SB203580 for 30 min before stimulation. After cell treatments, total RNA from two independent experiments was extracted, quantified and validated by Northern Blot (data not shown) prior to being converted to cDNA, labelled and hybridized with a mouse 44K whole genome microarray from Agilent. The microarray contained 41,175 probe-Ids representing 19,261 unique genes (Ensemble v52, *mus musculus *gene NCBIM37) with every gene being covered on average by at least two different probes. The hybridizations were referenced to a pool of cDNAs from two non-treated wild type MEFs used as a background control. The raw microarray and normalized data have been deposited in the Gene Expression Omnibus database (accession number GSE18320). To assess the statistical robustness of our microarray data we performed a Pearson correlation coefficient (PCC) analysis for all the probes found on the microarray using the statistical program R (version 2.10.0; http://www.r-project.org). All samples showed a high correlation coefficient between the two replicates with most correlation values higher than 0.9, indicating a high reproducibility between the experimental duplicates (additional file 1). The quality of the microarray data was also diagnosed using the Bioconductor package arrayQualityMetrics [15]. The quality of the microarray data was determined by MA plots whereas the reproducibility and homogeneity between the experiments was assessed respectively by Box plots and Density plots. All these analyses showed a high quality for both the array data and the experimental duplicates (additional files 2 and 6). Once we assessed the quality and reproducibility of the raw data we took the log2 ratio average of the two independent hybridizations per biological condition and subsequently the Agilent probe-Ids were converted to unique ensemble gene-Ids. On top of that, to minimize the false positives, we set up 2.5-fold induction cut off and considered those genes as true stress-induced genes and selected them for further analysis. The treatments with TNFα and anisomycin induced 2.5-fold a total of 144 and 146 unique genes, respectively, whereas NaCl induced 178 unique genes (Figure 2A). Although there is some gene overlapping between the treatments, each stimulus induced a large set of specific genes. The common response to the three treatments was limited to 30 unique genes (Figure 2B).

**Figure 3:**
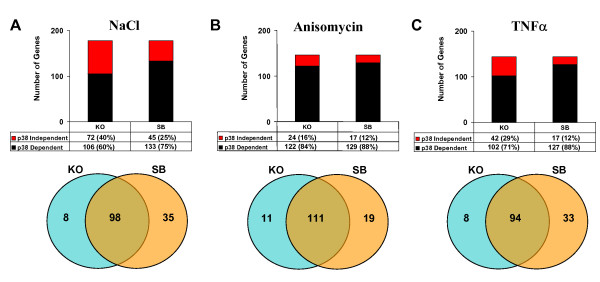
**p38 SAPK dependency of the genes up-regulated by anisomycin, TNFα and NaCl**. p38 SAPK dependency of the genes up-regulated more than 2.5 fold in wt MEFs pre-treated with SB203580 or in p38α^-/- ^MEFs treated with NaCl (A) anisomycin (B) or TNFα (C). The histogram shows the percentage of p38 dependent (black) and independent genes (red). Total p38 dependent (black) and independent (red) gene numbers are also shown. The Venn diagrams show the overlapping of p38 dependent genes between SB203580-treated wt MEFs and p38α^-/- ^MEFs.

We then analysed the contribution of p38α SAPK to the gene expression changes induced by different stimuli. To this end, we compared the gene expression profiles from wild type MEFs either with those obtained from p38α^-/- ^MEFs and from wild type MEFs treated with the p38α/p38β SAPK inhibitor SB203580. We considered that a gene was dependent on p38 SAPK activity when its expression was reduced at least 50% in SB203580 treated MEFs or in p38α^-/- ^MEFs. Taking this into account, the p38 SAPK dependency ranged between 60% and 88% depending on the treatments (Figure 3A-C, top histograms). Interestingly, the Venn diagrams (Figure 3A-C, bottom panels) showed in all cases a large degree of overlapping between the genes affected in p38α^-/- ^knock out MEFs and SB203580-treated MEFs, supporting that expression of these genes was indeed p38 dependent. A full list of the up-regulated genes and their p38 SAPK dependency is accessible as supplementary material in an Excel spreadsheet (additional file 3).

**Figure 4:**
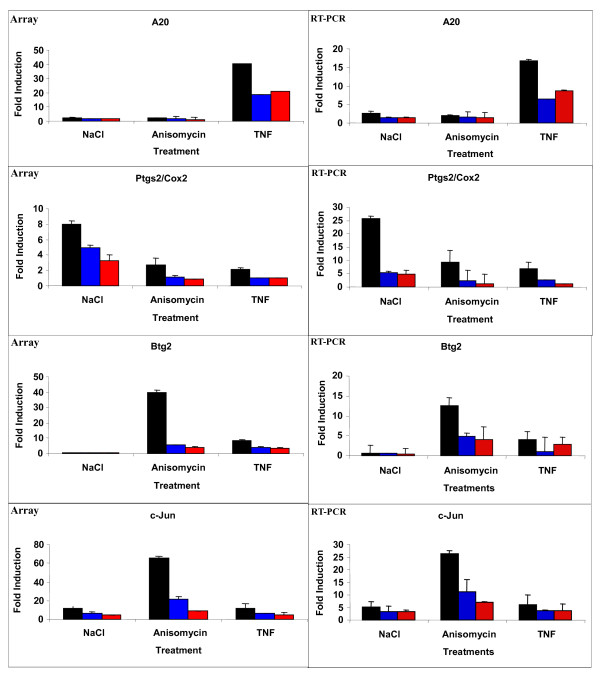
**Validation of the microarray data by RT-PCR**. The mRNA levels of 4 strongly up-regulated genes, A20, Ptgs2/Cox2, Btg2 and c-Jun, were determined by RT-PCR, corrected by the mRNA levels of GAPDH, quantified and plotted (right panels) alongside with the mRNA levels obtained from the microarrays (left panels). Black bars represent wt MEFs, blue bars p38α^-/- ^knock out MEFs and red bars wt MEFs pre-treated with SB203580. The average of two independent experiments is shown.

To validate our microarray analyses, we selected a few representative stress-regulated genes and tested their mRNA levels by RT-PCR. Notably, the tendency of regulation observed by RT-PCR confirmed the expression levels found in the microarrays. Thus, the TNFα cell signalling regulator A20 was only up-regulated by TNFα whereas the transcription factor c-Jun and the cell proliferation regulator Btg2 were mostly up-regulated by anisomycin. On the other hand, the pro-inflammatory gene Ptgs2/Cox2 was highly up-regulated by NaCl and to a lesser extent by anisomycin and TNFα. In all cases, the expression levels of the corresponding mRNAs were significantly reduced in both, SB203580 treated wild type MEFs and p38α^-/- ^knock out MEFs (Figure 4).

Activation of p38 SAPK by different stimuli also resulted in the down-regulation of some genes. Taking into account genes that were down-regulated at least 4 fold, the treatment with NaCl affected the expression of 22 genes, anisomycin 36 genes and TNFα 29 genes. Interestingly, the p38α dependency of genes down-regulated by NaCl ranged between 72 and 68%, while the genes down-regulated with anisomycin or TNFα were completely dependent on p38 SAPK (additional file 4), suggesting that the stress response down-regulates several genes and this pathway is also important in the process.

### Analysis of the function of induced genes shows major stimulus-specific responses and a restricted common response to all treatments

**Figure 5:**
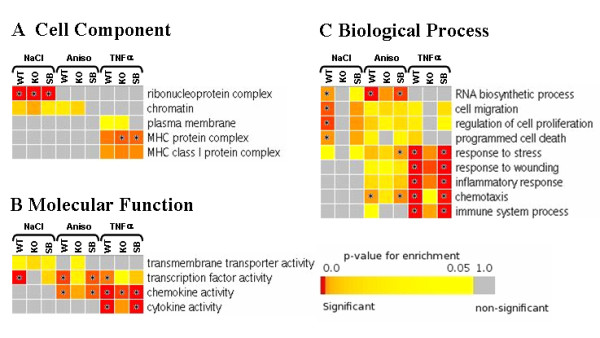
**GO analysis of the microarray data**. A. Heatmap statistics showing significantly (p-value ≤ 0.05) enriched GO cell component categories among the gene expression changes induced by NaCl, anisomycin and TNFα. The p38 SAPK involvement in the category is assessed by comparison with the affected genes found in p38α^-/- ^knock out MEFs and SB203580-treated wild type MEFs. Colours toward red indicate high statistic significance, yellow indicates low statistic significance, and gray indicates no statistic significance. Star marks the tests that are significant after false discovery rate correction (FDR q value ≤ 0.05). Full statistics are shown in the supplementary additional file 5. B. Molecular function GO analysis among the three treatments, as in A. C. Biological process GO analysis among the three treatments, as in A.

To better understand the role of the genes induced in response to each stimulus, we have performed a gene set enrichment analysis based on the gene ontology (GO) categories corresponding to *cellular component*, *molecular function*, and *biological process *[16] as described in Methods. The *cell component *enrichment revealed that NaCl up-regulated in a p38 SAPK dependent manner many genes encoding members of the ribonucleoprotein complex, including several proteins of the large and small ribosome subunit. In addition, NaCl and anisomycin but not TNFα up-regulated DNA associated proteins such us the histone variants H1fo, H1d, H1t, H1.2, H2a type f, novel H2b, H2A type 3, H3.f3a and H3.3b. On the other hand, TNFα induced genes encoding many plasma membrane proteins, including components of the major histocompatibility complex such us H2-OB, H2K1, H2Q1, H2Q10 and B2 m (Figure 5A and additional files 5 and 6).

Furthermore the analysis for enrichment of the *molecular function *showed that NaCl specifically up-regulated genes encoding for trans-membrane transporters belonging to the solute carrier family of transporters like Slc15a1, Slc35f1, Slc6a1, Slc5a1, Slc22a3, Slc20a1 and Slc38a2. Interestingly among this group of genes we also found several ion trans-membrane transporters like Accn2, Trpm3 and Kcnv2. Notably the main enriched *molecular function *induced by the three treatments was the up-regulation of a set of genes belonging to the transcription factor activity GO term. NaCl up-regulated 24 transcription factors while anisomycin and TNFα up-regulated 17 and 19 transcription factors respectively. Each treatment specifically up-regulated an specific set of transcription factors. For instance, NaCl induced the expression of Mafk, n-Myc, Foxg1, Foxc1, Hoxc12 and Runx2, whereas anisomycin induced the transcription factors Fosb, Sox2, Smad7, Ddit3, Klf11 and Egr2. On the other hand TNFα induced Nfatc1, Pax1, Lhx5, Gsc, Nkx2-9, Fosl1 and Irf1. Thus, the differential regulation of transcription factors seems to be a key process to build up the appropriate gene response for cell adaptation to a particular stress. It is worth noting that, in contrast to NaCl and TNFα, anisomycin also induced a set of genes belonging to the Id family of transcription repressors, such as Id1, Id2 and Id4. In addition, TNFα and anisomycin also enhanced genes belonging to the GO term *chemokine activity*. As expected, these genes were also induced in cells treated with the pro-inflammatory TNFα cytokine but the overall response was less prominent in anisomycin-treated cells. For instance anisomycin up-regulated the chemokines CCL4/20/22 and Cxcl10 whereas TNFα also induced the chemokines CCL2/7, CXCL1/2/3/5/9, and the cytokines Csf1, Spp1, Tslp and GDF10. Taken together, our data indicate that anisomycin partially elicits a TNFα mediated response (Figure 5B and additional files 5 and 6).

Finally, we analysed for the *biological process *GO term and found that NaCl and anisomycin strongly up-regulated genes involved in the RNA biosynthesis process. Moreover, the three stimuli were able to induce the processes of cell migration, cell proliferation, programmed cell death and, as expected, response to stress. However the response to stress induced by NaCl was milder after 2 h of stress compared to that elicited by anisomycin or TNFα, which up-regulated a large set of genes. Moreover, TNFα clearly promoted the up-regulation of genes implicated in the responses to wounding and inflammation as well as in the immune response and chemotaxis. Anisomycin also up-regulated genes related to these biological processes, although to a lesser extent than TNFα, indicating that this compound somehow partially mimics TNFα action (Figure 5C and additional files 5 and 6). A full list of the genes found in each category is presented in the supplementary web file (additional file 6)

As previously reported activation of the p38 pathway promotes its down-regulation through the expression of Dual Specificity Phosphatase (DUSP) genes [17]. Although the induction of this particular biological process was not found to be statistically enriched among the three treatments, this is due that different stimuli induced different sets of DUSP genes. Thus, TNFα induced DUSP1, DUSP8 and DUSP16, anisomycin induced DUSP1, DUSP8, DUSP10 and DUSP16, whereas NaCl induced DUSP1, DUSP4 and DUSP10. Therefore, p38 SAPK activation contributed to different extend to DUSP gene up-regulation. The up-regulation of these phosphatases might differentially contribute to the kinetics of the stress response mediated by a particular stimulus.

To further mine the microarray data, we performed an Ingenuity Pathway Analysis (IPA; http://www.ingenuity.com) that allows us to infer the most relevant gene networks controlled by p38 SAPK signalling. According to the *molecular functions *and *biological processes *mentioned above, the most relevant gene network induced by TNFα was related to the immune response and immunological disease functions (additional file 7). Instead, the anisomycin gene network was related to the regulation of cell development and gene expression. The main transcription factors induced were c-Fos, Jun, Nfatc2, Ddit3, Sox2, Maff and Id2 (additional file 8). Osmostress induced a gene network that is related to the control of cell cycle and posttranslational modification with the c-Fos, Egr1, FoxG and PRRX1 as the main transcription regulators (additional file 9).

**Figure 6:**
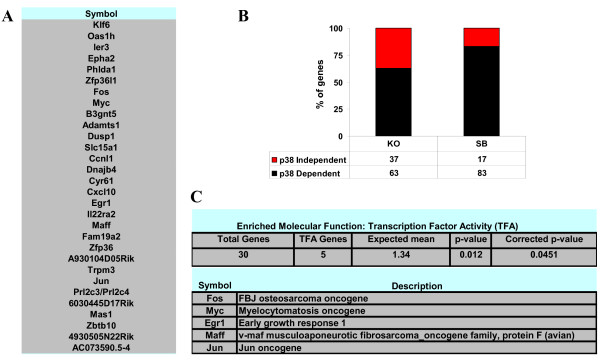
**Identification of a common gene response induced by the 3 treatments**. A. The 30 common genes up-regulated more than 2.5 fold by NaCl, anisomycin and TNFα is shown. B. Histogram showing the percentage of p38 SAPK dependent (black bars) and independent (red bars) genes. We have considered a gene is p38 SAPK dependent when its expression is reduced at least by 50% in two independent treatments. C, Binomial statistics showing that transcription factor activity is the most significant GO molecular function enriched among the 30 common genes. The five common up-regulated transcription factors are shown.

As mentioned above, all three treatments strongly induced a core of 30 common genes which expression showed a high dependency on p38 SAPK activity (Figure 6 and additional file 4). Overall, the p38 SAPK dependency ranged from 63% in p38α^-/- ^cells to 83% in SB203580 treated wild type MEFS. Interestingly, 5 out of these 30 genes are common transcription factors, namely c-Fos, N-myc, Jun, Egr1 and Maff (figure 6C), which represents a significant enrichment for this functional category. Due to the relative small number of common genes, we could not identify any trend on p38 SAPK independent genes. For those p38-dependent genes, the IPA software allowed us to uncover the most relevant common gene networks which are those mainly involved in the control of gene expression, cell cycle and cell fate (additional file 10).

### Kinetic analysis of p38 SAPK-mediated gene expression in response to osmostress

Little is known on the kinetics of gene expression in response to p38 SAPK activation. To shed some light on how this pathway regulates gene transcription in a time dependent manner, MEFs cells were subjected to osmostress (100 mM NaCl) in the absence or the presence of the SAPK inhibitor SB203580. Cells were collected at 45 minutes, 2 h and 8 h upon osmostress and RNAs were subjected to microarray analysis as before. As described above, the average of two independent hybridizations was taken and the probe-Ids were converted to unique ensemble gene-Ids. Genes up-regulated at least 2.5-fold were selected for functional analyses.

**Figure 7:**
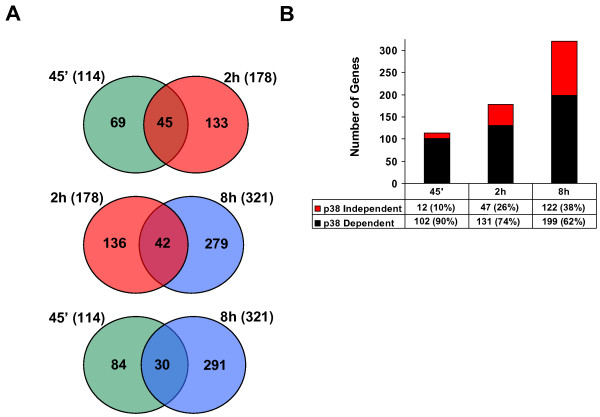
**Genes up-regulated at different times upon NaCl treatment and their dependency on p38 SAPK**. A. Venn diagrams show the total numbers of genes up-regulated more than 2.5 fold at 45 minutes, 2 h and 8 h upon NaCl treatment and the overlapping between two given times. B. The histograms show the p38 SAPK dependency of the genes up-regulated more than 2.5 fold for the indicated times. The histogram represents the total p38 SAPK dependent (black) and independent (red) gene numbers. The percentage of p38 SAPK dependent (black) and independent (red) genes are shown.

As shown in figure 7A, a total of 114, 178 and 321 genes were up-regulated 2.5-fold upon osmostress at 45 minutes, 2 h and 8 h, respectively. A full list of up-regulated genes is provided as supplementary material in an Excel spreadsheet (additional file 3). The overlapping among different time-points was restricted to a subset of genes, being most of them specifically up-regulated in a time dependent manner. It is worth noting that the p38 SAPK dependency for the osmostress-responsive genes decreased over time. Thus, 90% of the genes up-regulated by NaCl at 45 minutes were affected by SB203580, whereas this percentage dropped to 74% at 2 h and 62% at 8 h after salt addition (Figure 7B). Therefore, the role of p38 SAPK in the regulation of gene expression appears to be critical for the early response to stress, whereas other signaling pathways also become important at later stages of the adaptation process.

**Figure 8:**
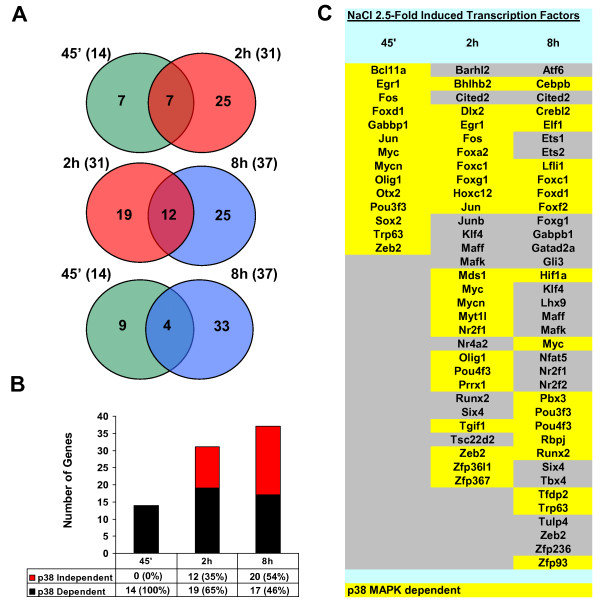
**Transcription factors up-regulated at different times upon NaCl treatment and their dependency on p38 SAPK**. A. Venn diagrams show the total numbers of transcription factors up-regulated more than 2.5 fold at 45 minutes, 2 h and 8 h upon NaCl treatment and the overlapping between two given times. B. The histogram represents the total numbers of p38 SAPK dependent (black) and independent (red) transcription factors for the indicated times. The percentages of p38 SAPK dependent (black) and independent (red) transcription factors are shown. C. A full list of 30 transcription factors up-regulated more than 2.5 fold by NaCl at the indicated times, and their p38 SAPK dependency, as determined by SB203580 treatment of wt MEFs.

A major *molecular function *controlled by stress is the up-regulation of genes encoding for transcription factors. Thus, we assessed the number of transcription factors that were specifically up-regulated by NaCl in a time dependent manner and found that they increased over time. Osmostress induced 14, 31 and 37 transcription factors at 45 minutes, 2 h and 8 h, respectively (Figure 8A). The overlapping among the three time points of the study was restricted to a subset of transcription factors. Thus, 7 transcription factors were up-regulated at 45 minutes of NaCl treatment, and 25 transcription factors were up-regulated at 2 h and 8 h, respectively. We also detected 4 transcription factors that were induced at 45 minutes and 8 h, but not at 2 h. Although a cyclic expression pattern cannot be ruled out, a most plausible explanation would be that the probe hybridization failed at the middle time-point and that those transcription factors were in fact up-regulated very early and remained high till later times. Interestingly, all the transcription factors up-regulated at 45 minutes upon osmostress were dependent on p38 SAPK activation. However, the p38 SAPK dependency decreased to 65% and 46% at 2 h and 8 h, respectively, after NaCl addition (Figure 8B). These data suggest that the primary wave of up-regulated transcription factors plays a key role on cell adaptation to osmostress and it is mainly driven by the activation of the p38 SAPK. In addition, other osmostress-activated signalling pathways are activated at later times to contribute to stress adaptation. A comprehensive list of the up-regulated transcription factors and its p38 SAPK dependency is shown in Figure 8C.

**Figure 9:**
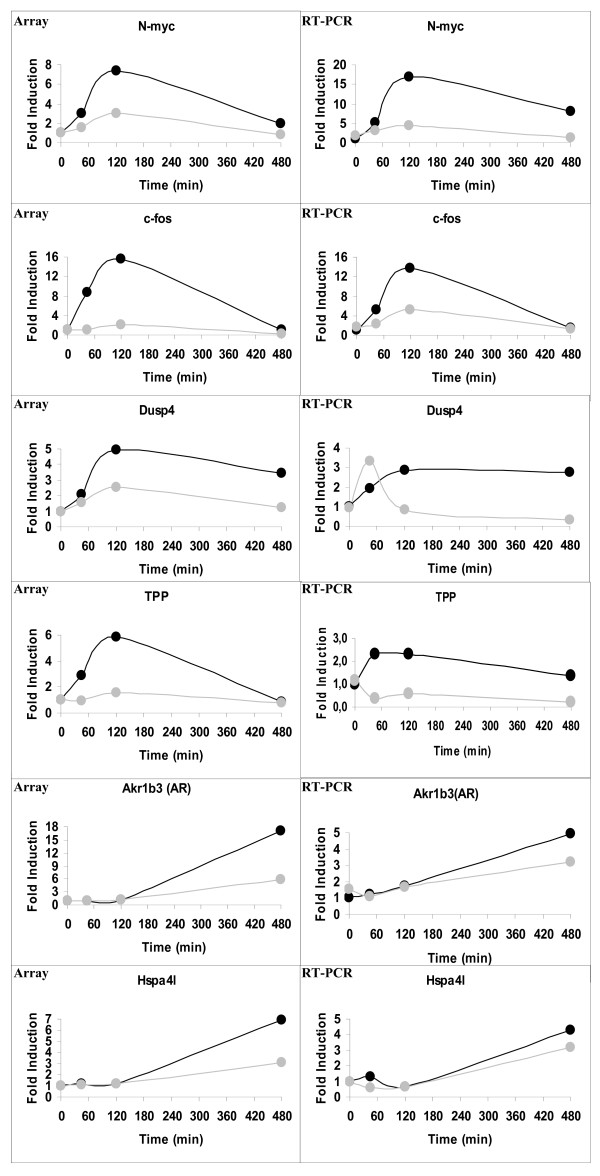
**Validation of the NaCl time-course by RT-PCR**. The mRNA levels of 6 strongly up-regulated genes, N-myc, c-fos, Dusp4, Tpp Akr1b3 (AR) and Hspa4l, were determined by RT-PCR and corrected by the mRNA levels of GAPDH, quantified and plotted (right panels) alongside with the mRNA levels obtained from the microarrays (left panels). Black circles indicate wt MEFs and grey circles wt MEFs pre-treated with SB203580. The average of two independent experiments is shown.

We validated our microarray analyses monitoring selected osmostress regulated genes by RT-PCR. The transcription factors n-Myc and c-Fos, the dual specifity phosphatase DUSP4 and the mRNA binding protein TPP were all highly up-regulated between 45 minutes and 2 h after osmostress and their mRNA levels dropped at the 8 h time point with the exception of DUSP4 which levels remained constant between 2 h and 8 h after NaCl addition. On the other hand, the aldo-keto reductase Akr1b3 (AR) and the heat shock protein Hspa4l genes were up-regulated later and their mRNA levels increased after 8 h of osmostress. In addition, the mRNA level of all these genes was clearly reduced in the presence of the inhibitor SB203580 confirming that their expression was dependent to some extent on the p38 signalling pathway (Figure 9).

### Gene Ontology analysis of p38 SAPK-mediated gene expression in response to osmostress

**Figure 10:**
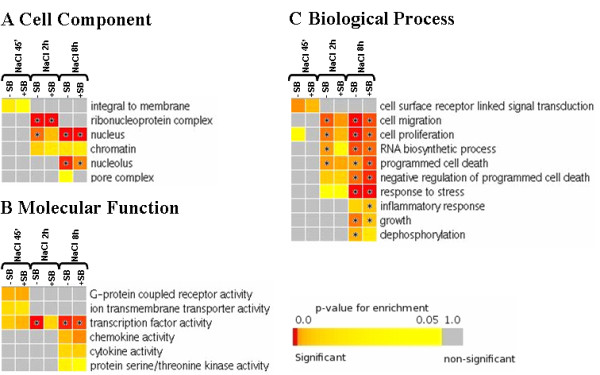
**GO analysis of the NaCl time course**. A. Heatmap showing significantly (p-value ≤ 0.05) enriched GO cell component categories after NaCl addition at the indicated times. The p38 SAPK involvement in the category is assessed by comparison with the affected genes found in SB203580 treated wt MEFs. Colours toward red indicate high statistic significance, yellow indicates low statistic significance, and gray indicates no statistic significance. Star marks the tests that are significant after false discovery rate correction (FDR q value ≤ 0.05). Full statistics are shown in the supplementary additional file 5. B. Molecular function GO analysis among the three times of study, as in A. C. Biological process GO analysis among the three times of study, as in A.

To gain additional insights into the function of osmostress induced genes in addition to the transcription factors, we performed a comparative GO gene enrichment analysis as described above at 45 minutes, 2 h and 8 h upon osmostress. We focussed the analysis in the most relevant gene categories up-regulated upon osmostress and we took into account the p38 SAPK dependent genes. The GO term *cell component *showed that most of the genes induced by p38 SAPK at 45 minutes encode for proteins that function at the plasma membrane. However, after 2 h of osmostress many up-regulated genes encode proteins that function in the ribonucleoprotein complex or are located in the cell nucleus and the chromatin. These two *cell locations *were also enriched 8 h after salt addition. At 8 h, we also found enrichment in some other sub-nuclear locations, namely the nucleolus and the nuclear pore complex (Figure 10A and additional files 5 and 6).

The main p38 SAPK dependent *molecular function *elicited by osmostress at 45 minutes is related to G-protein-coupled receptor activity and ion trans-membrane transporters. As described above, the *transcription factor activity *GO term was considerably enriched at 45 minutes and increased progressively over time after NaCl addition. On the other hand, at later times p38 induced chemokine and cytokine activities as well as protein serine/threonine kinase activity (Figure 10B and additional files 5 and 6). Importantly, long exposure to osmostres also up-regulated several serine/threonine kinases (Stk4, Plk2, Cdc2l5, PKCα or Sgk1) some transmembrane protein tyrosine kinase receptors (Epha2, EGFr or VEGFr), Src-like tyrosine kinases (Lyn) and dual specificity serine/tyrosine kinases (Styk1).

The third GO analysis performed gave us information on the *biological processes *regulated in a p38 SAPK- in a time-dependent manner. As shown in figure 10C, the major up-regulated *biological process *at 45 minutes was the surface-linked signal transduction. This observation is in full agreement with the main *cell location *and *molecular function *of the genes found up-regulated at that time. Moreover from 2 h after the addition of NaCl we found enrichment of genes involved in the processes of cell migration, cell proliferation, RNA biosynthesis, negative regulation of programmed cell death and response to stress. Finally long exposure to NaCl induced the up-regulation of a different set of genes involved in the control of the inflammatory response, cell growth and protein dephosphorylation (Figure 10C and additional files 5 and 6). The last process is partly related to the down-regulation of MAPK activity. For instance, osmostress specifically induced several DUSPs at different times. DUSP1 was up-regulated at 45 minutes and their levels remained high till 8 h after osmostress. After 2 h of NaCl addition, DUSP4 and DUSP10 were up-regulated and the expression levels of DUSP4 remained high till the 8 h time-point. On the other hand, DUSP14 was specifically up-regulated at 8 h. On top of that, several tyrosine phosphatases were upregulated at 8 h of osmostress (PTPLA, PTP4A1, PTP4A2, PTPRB and PTPRD) as well as the serine/treonine phosphatase PPMD1. Altogether, the up-regulation of phosphatases suggests that this is an important event to down-regulate osmostress-induced signalling pathways.

It should be noted as well, that long exposure to NaCl induced a strong *stress *and *defence response *which resembles the TNFα mediated response. Specifically the chemokine/cytokines CCL7/20, CXCL3/5, Spp1 and Pdgfb were induced after 8 h of NaCl treatment. All these chemokines, except Pdgf1b, were up-regulated by TNFα as well, while only CCL20 was up-regulated by anisomycin. The up-regulation of chemokines and cytokines indicates that long exposure to NaCl induces a late TNFα-like response necessary for cell adaptation to osmostress. A full list of genes found in each category can be accessed through the supplementary miniweb file (additional file 6).

## Discussion

The activity of p38 SAPK is important for controlling many aspects of cell physiology and its role in gene expression regulation has been well established. For instance, the *Saccharomyces cerevisae *p38 SAPK orthologue Hog1 plays a key role regulating osmostress-induced genes required for yeast adaptation to osmotic shock [3]. In order to shed light on the role of p38 SAPK in genome-wide transcriptional regulation in response to stress, we have performed whole genome microarray analyses and compared three different treatments (TNFα, anisomycin and NaCl) that are able to activate p38 SAPK, albeit with different kinetics. The three p38 SAPK activators induced the up-regulation of a specific set of genes, with a small core set of genes being induced by all of them.

In general, genes induced by the three stimuli were found to be involved in the regulation of different biological processes. As expected, TNFα elicited immune and stress responses. On the other hand, anisomycin specifically induced genes involved in transcription repression and chemokine activity suggesting that this antibiotic partially mimics TNFα, which strongly induced several chemokines and cytokines. Both NaCl and anisomycin had in common the regulation of RNA biogenesis whereas NaCl specifically enhanced many components of the ribonucleoprotein complex, suggesting that protein translation is a key process for cell adaptation to osmostress. On top of that, osmostress also up-regulated DNA associated proteins, such as histones and proteins associated to the nucleolus and the nuclear pore complex. Notably, the three stimuli regulated to some extent genes involved in cell migration, proliferation and cell death. However, the major GO *molecular function *induced by p38 SAPK activation was the up-regulation of transcription factors.

A significant amount of up-regulated genes was dependent on p38 SAPK, based on their diminished expression in the presence of SB203580 and in p38α^-/- ^cells. This raises the interesting question of how SAPK activity is able to specifically up-regulate different gene programs depending on the stimuli. A plausible explanation would be that the final output results from the integration at the gene promoters of various converging signals. Thus, the presence of additional activating signals would be necessary to achieve the highest transcriptional up-regulation in a given context, while inhibition of p38 SAPK would cut one of the inputs resulting in a reduced expression. Alternatively, but less likely, the different kinetics observed in the activation of p38 SAPK might lead to the activation of a different set of genes.

It is well established as well, that p38 SAPK controls the stability of several mRNAs through a not fully understood mechanism that requires the activity of MK2 and the presence of AU-rich elements (AREs) located at the 3' UTR of specific mRNAs. These sequences are involved in the recruitment of ARE-binding proteins that contribute to mRNA stabilisation or degradation [18]. There are about 950 human mRNAs containing ARE sequences. A high-throughput study from Frevel and co-workers showed that the stability of up to 42 mRNAs bearing ARE sequences were enhanced upon the treatment of the human monocytic cell line THP-1 with lipolysaccharide in a p38 SAPK dependent manner [19]. We have crossed Frevel's results with ours and found the overlapping of up to 12 different genes (e.g. Tnfaip3, Gro1, Gro2, Gch1, PTGS2/COX2, Ccl2, Cxcl10, Junb, Csf1, Irf1, Pmaip1 and Cited2). Notably, most of these genes where up-regulated by TNFα which suggests that different p38 SAPK activators may contribute differently to mRNA stabilization. Moreover, we have seen that osmostress strongly up-regulated the ARE binding protein TPP which has been shown to suppress inflammation by accelerating the decay of cytokine mRNAs upon p38 SAPK activation [20]. Therefore further experimental work is necessary to asses the contribution of transcription and mRNA stabilization in response to stress conditions. In any case, p38 SAPK appears to be instrumental for the proper response of a majority of genes in response to all three stimuli tested.

In yeast, there is a large conserved environmental stress response (ESR), which consists in a core of genes that respond to a diverse type of stress; such as heat shock, osmostress, oxidative stress and others [21,22]. This core of genes includes a number of stress defence genes and seem to be important for adaptation to stress. In mammals, such an ESR has not been reported. Interestingly, we found a small number of genes that is common to all three stress treatments and might be relevant. Notably, the main enriched *molecular function *induced by the three treatments was the up-regulation of transcriptional modulators. Thus, transcriptional regulation could be a key shared process to build up the appropriate gene response for adaptation to cell stress. Moreover, the three treatments up-regulated several members of the DUSP family of genes, which have MAPK phosphatase activity and are known to play a key role in the down-regulation of MAPK signalling [23,24].

It is worth noting that when p38 SAPK dependence was analysed in a time course experiment, more than 90% of the genes that responded to osmostress at 45 minutes were p38 SAPK-dependent, however, after 8 h only 60% of the up-regulated genes required p38 SAPK activity. Thus, the SAPK pathway appears to be critical at the initial response, whereas other signaling pathways are probably more relevant at later times.

Regarding how cells adapt to stress over time, our GO analyses revealed that the primary response to NaCl addition was the up-regulation of solute and ion trans-membrane transporters as well as sensorial G-protein coupled receptors. Significantly, 65% of the early genes (71 out of 114 up-regulated at 45 minutes) were shut down at later times. At the intermediate time point, 130 genes were specifically up-regulated and were mainly involved in the control of transcription and ribosome biogenesis. However after 8 h of osmostress, 279 genes were specifically up-regulated. These late-induced genes were mainly included in the GO terms transcription factor activity, chemokine and cytokine activity, protein kinase activity, stress response, growth control and protein dephosphorylation. Altogether, we conclude that on one hand, osmostress up-regulates many ion trans-membrane transporters to quickly overcome the changes in osmolarity between the extra-cellular environment and the intracellular milieu, whereas on the other hand, long exposure to osmostress promotes cell survival, growth and mediates a TNFα-like response through the up-regulation of chemokine/cytokine activities. On top of that, protein dephosphorylation is probably necessary to down-regulate p38 SAPK activity and to shut down other signalling pathways activated at early times. Notably, gene transcription is still necessary for cell adaptation at this late time point.

To date, few whole genome studies on p38 SAPK regulated genes have been reported. An early work used primary endothelial cells treated with TNFα in the presence of SB203580 for 5 h to identify TNFα-induced and p38 SAPK dependent genes. These authors reported 58 TNFα-induced genes, 12 of which depended on p38 SAPK to some extent [10]. In contrast, we have found that just after 45 minutes of incubation with TNFα there is a strong dependency on p38 SAPK for gene up-regulation. This difference would be consistent with our results showing that SAPK dependency decreases over time upon osmostress treatment. Therefore, the genes up-regulated 5 h after cell stimulation observed by Vienmman and co-workers may represent a late response to the cytokine, which would be driven by secondary signalling events rather than by TNFα itself. In agreement with this idea, our data shows that TNFα strongly induced the up-regulation of chemokines and cytokines that once secreted to the media may trigger a paracrine cell response leading to the activation of other signalling pathways and gene transcription programs. Similarly a more recent study reported a genome wide screening using rat fibroblast-like synoviocytes also treated with TNFα. These authors reported the up-regulation of 141 genes by this cytokine, 30% of which were dependent on the activation of p38 SAPK [11]. Again the low p38 SAPK dependency reported in this work is not surprising if we take into account that cells were harvested 24 h after the addition of TNFα. We have crossed-checked their TNFα up-regulated list of genes with ours and found that only six genes overlapped: Cxcl2, Gm190, Csf1, Pde4b, Phlda1 and Ier3. As discussed above, the most plausible explanation for the discrepancy with our study is probably due to the fact that we have evaluated the primary TNFα response on immediate early genes whereas Zer *et al *reported a late TNFα-mediated gene response. On another hand, a wide genome microarray analysis performed on exponentially growing human keratinocytes treated with SB203580 but without challenging the cells with extra-cellular stimuli has been reported. This study concluded that p38 SAPK positively controlled genes involved in cell proliferation, keratinocyte differentiation and MAPK regulation [12]. In agreement with our results, the authors also found the up-regulation of some DUSP genes, indicating that this is likely to be an important event to down-regulate MAPK activity. Moreover Gazel's work and ours concludes that the transcription factors c-fos and n-Myc are key players of stress-mediated responses. However, the overall gene overlapping between the set of genes that we have found and that of Gazel and co-workers is negligible which might be due to the fact that in such study the authors only evaluated gene regulation mediated by the p38 SAPK basal activity. Our conclusions based on transient activation of the p38 SAPK also differ from a report using proliferating cardiomyocytes which showed that p38 SAPK basal activity played an important role in regulating extra-cellular matrix genes through the transcription factors C/EBPβ and TEF-1 [13].

## Conclusions

Our work illustrates how acute and transient p38 SAPK activation control gene expression in response to different stresses. It appears that the primary transcriptional response induced by three stimuli is highly dependent on p38 SAPK activation, but then becomes diluted over time. As expected, TNFα induced a strong pro-inflammatory response. Anisomycin, on the other hand, partially elicited a response that resembles a mild TNFα mediated response. At early times, NaCl clearly up-regulated many trans-membrane transporters probably to compensate the strong change of osmolarity between the cell and the extra-cellular environment. At the middle time point, osmostress strongly enhanced the expression of many genes involved in ribosome biogenesis suggesting that *de novo *protein translation is fundamental for cell adaptation. At this time, we also start to see how the cells respond to stress through the up-regulation of some chemokines. This is further strengthened at later times where NaCl induced a strong defence response. In addition, the osmostress-induced up-regulation of trans-membrane transporters, ribosome proteins and histones had not been previously shown. Moreover, we have defined a core of 30 common genes, five of which are transcription factors, which are regulated by several stresses. Therefore the regulation of transcriptional activity emerges as the most prominent molecular function depending on p38 SAPK activation that is induced by the three stress stimuli.

## Methods

### Cell culture and treatments

Immortalyzed wild type and p38α^-/- ^mouse embryonic fibroblasts (MEFs) [25] were maintained in Dulbecco's modified Eagle's medium (Biological Industries) containing 10% fetal calf serum (Sigma) and supplemented with 1 mM sodium pyruvate, 2 mM L-glutamine, 100 U/ml Penicilin and 100 μg/ml Streptomycin (GibCO) and cultured in a 5% CO_2 _humidified incubator at 37°C. MEFs were treated with 100 mM NaCl for 45 minutes, 2 h and 8 h, 25 ng/ml anisomycin (Sigma) for 45 minutes or 100 ng/ml TNFα (Preprotech) for 45 minutes. When indicated, cells were treated with 10 μM SB203580 (Calbiochem) for 30 minutes prior to the treatments.

### RNA preparation and microarray analysis

Total RNA was isolated from exponentially growing wt MEFs and p38α^-/- ^MEFs using the RNeasy kit according to the manufacturer's protocol (Qiagen, Dusseldorf, Germany). RNA amplification and labeling was carried out by using the Low RNA Input Linear Amplification Kit, PLUS, Two-Color (Agilent Technologies, Palo Alto, CA). Briefly, for each sample 2 μg of total RNA input were amplified in two rounds of amplification by following manufacturer's instructions. The first strand cDNA syntheses and amplification reactions were carried out by using random and T7 primers, respectively. During the 2 hour *in vitro *transcription, Cy3- or Cy5-labeled CTP was incorporated on each amplified RNA (cRNA) from reference pooled and tested samples respectively. Products of the reaction were then purified using RNAeasy mini spin column (Qiagen, Dusseldorf, Germany). All procedures of hybridization, slide and image processing were carried out according to the manufacturer's instructions (Two-Color Microarray-Based Gene Expression Analysis protocol). In each experiment, 825 ng of contrasting cRNA samples were fragmented at 60°C for 30 min and hybridized at 65°C for 17 hr. The slides were scanned at a 10 μm resolution with Agilent G2565BA Microarray Scanner (Agilent Technologies, Palo Alto, CA). Signal quantification was carried out with Feature Extraction 9.1 software (Agilent Technologies, Palo Alto, CA) by using default analysis parameters for Agilent's 44K whole genome mouse gene expression arrays (Feature Extraction protocol 44K). The reproducibility of the microarray data was assessed by performing a Peason Correlation Coefficient Analysis (PCC) of the two biological replicates using the statistical computing program R (version 2.10.1; http://www.r-project.org/) between the absolute signal intensity of the background corrected Cy5 channel (treated samples) and the absolute signal intensity of the background corrected Cy3 channel (control samples) for all the probes present on the microarray. The quality and homogeneity of the background corrected and VSN normalized microarray data [26] for the Cy5 and Cy3 channels was assessed by the Bioconductor package arrayQualityMetrics [15] version 2.4.3 http://bioconductor.org/packages/2.5/bioc/html/arrayQualityMetrics.html under R version 2.10.1 http://www.r-project.org/.

### RT-PCR validation

Total RNA was converted into cDNA with the Superscript First-Strand kit (Invitrogen). PCR primers for individual genes selected after the microarray analysis were designed with Primer3 [27] to generate DNA fragments between 100-200 base pairs in length and a Tm of 56°C. The sequences used are the followings: A20 For: CATGTCGACGTCGAGACCATGGCTAAC, A20 Rev: CATGTCGACCATTGGTTTTCAGAGCCACG, Ptgs2/Cox2 For: TACAAGCAGTGGCAAAGGC, Ptgs2/Cox2 Rev: CAGTATTGAGGAGAACAGATGGG, Btg2 For: ACGCACTGACCGATCATTACA, Btg2 Rev: GGCTGGCTGAGTCCAATCTGG, c-Jun For: CCCCTGAGAACGACGCAAGCC, c-Jun Rev: GATGAACAGTCCGGAGTCCGCG, Gapdh For: AATTCAACGGCACAGTCAAGGC, Gapdh Rev: GGATGCAGGGATGATGTTCTGG, n-Myc For: CATGCCGGGGATGATCTGCA

n-Myc Rev: TCGAATTGGGCTACGGAGAT, Dusp4 For: GTCTCTGGACCCCAAATCCA, Dusp4 Rev: CAATAACGGCGGTTTCCGCT, Ttp For CTCTGCCATCTACGAGAGCC, Ttp Rev GATGGAGTCCGAGTTTATGTTCC, c-Fos For: GGCTTTCCCAAACTTCGACC

c-Fos Rev: GGCGGCTACACAAAGCCAAAC, Hspa4l For: TCGGCTTTCTCAACTGCTAC, Hspa4l Rev: CTTCCAGGTACCGCACCTTA, Akr1b(AR) For: ATGGCCAGCCATCTGGAACTC, Akr1b(AR) Rev: CACACCCTCCAGTTCCTGTT

### Enrichment Analysis of Gene Ontology and Gene Network Analysis

Functional annotation of genes is based on Gene Ontology (GO) [16] as extracted from Ensembl v.52 [28]. Accordingly, all genes are classified into three ontologies: genes involved in Biological Process, Molecular Function and Cellular Component. We have taken only the GO categories that have at least 20 genes annotated. In our study we distributed up-regulated genes defined as genes with at least 2-fold increase upon cell stimulation for analysis of Cellular Component and 2.5 fold increase for analysis of Molecular Function and Biological Process in GO-terms and observed which terms are enriched using GiTools (http://www.gitools.org, manuscript in preparation). In search of statistical significance (p-value) we used binomial distribution and p-value calculated as:

Where:

***n ***= total no. of genes in the category

***x ***= number of differently expressed genes in the category.

***p ***= frequency of differentially expressed (upregulated or downregulated) genes

Resulting p-values were adjusted for multiple testing correction using the Benjamin and Hochberg's method of False Discovery Rate [29]. Colours toward red indicate high statistic significance. Colours toward yellow indicate low statistic significance and gray indicates no statistic significance at all. Significant gene networks were extracted from 2.5-fold up-regulated genes using the Ingenuity Pathway Analysis (IPA) tool developed by Ingenuity^R ^Systems http://www.ingenuity.com

### Western Blot

Cells were washed with ice-cold PBS and scraped into 500 μl of IP/lysis buffer (10 mM Tris HCL pH 7.5, 1% NP40, 2 mM EDTA, 50 mM NaF, 50 mM β-glycerophosphate, 1 mM Sodium Vanadate, supplemented with the protease inhibitors 1 mM PMSF, 1 mM Benzamidine, 200 μg/ml Leupeptine and 200 μg/ml Pepstatine). The lysates were cleared by microcentrifugation. The primary antibodies used for these analyses were rabbit anti-p38α, rabbit monoclonal anti-pp38T180/T182, rabbit anti-MK2, anti-ppMK2 (Cell Signaling) and mouse mAb anti-tubulin (Sigma). For visualizing the proteins, Horse Radish Peroxidase conjugated anti-rabbit or an anti-mouse and the Enhanced Chemiluminiscence kit (GE Healthcare) were used.

## Authors' contributions

IF, MB and MJ performed the biological experiments. LL and OD performed the microarray hybridization. AI and NL-B performed the PCC, arrayQualityMetrics and GiTools analysis. GG-L and DP performed the Ingenuity Pathway analysis. Experimental design, biological data interpretation and manuscript writing was carried out by MJ, AN and FP. All authors read and approved the final submission.

## Supplementary Material

Additional file 1 **Pearson correlation coefficient analysis (PCC)**. Correlation between the two replicates was analyzed by PCC as described in methods. All samples showed very high correlation coefficient with most correlation values higher than 0.9, which indicates high fidelity of the experimental biological replicates. Click here for file

Additional file 2 **Microarray Quality Metrics Analysis**. For a better visualization the microarray data the diagnosis has been split in three groups named 1; Treatment and Time Course. 2; p38α SAPK knock out MEFs and 3; SB203580 treated wildt type MEFs. Section 1 analyses the individual array quality through MA. The mass distribution in an MA plot is expected to be concentrated along the M = 0 axis and there should be no trend in the mean of M as a function of A. A trend in the lower range of A usually indicates that the arrays have different background intensities. A trend in the upper range of A usually indicates saturation of the measurements. Section 2 analyses the array intensity distributions through Box plots and Density plots. Each Box box corresponds to one array. Array duplicates are shown side by side. The left panel corresponds to the red Cy5 channel. The middle panel corresponds to the green Cy3 channel. The right panel shows the Box plots of the log_2 _Cy5/Cy3 ratio. Box plots are a graphical representation that summarises the distribution of probe intensities across all arrays. It comprises the smallest observation, lower quartile, median, upper quartile and largest observation. All boxes are expected to have similar size and median. Density plots show smoothed histograms of the array signal intensities. The left panel corresponds to the red Cy5 channel. The middle panel corresponds to the green Cy3 channel. The right panel shows the Box plots of the log_2 _Cy5/Cy3 ratio. The distribution of the arrays is expected to have similar shapes and ranges. Click here for file

Additional file 3 **2.5-fold up-regulated genes upon transient p38 SAPK activation**. List of unique Ensemble-Id genes up-regulated 2.5 fold over a pool of control cells upon a transient p38 SAPK activation. Wild type MEFs and p38α^-/- ^knock out MEFs cells were stimulated with 100 ng/ml TNFα, 25 ng/ml anisomycin and 100 mM NaCl for the indicated times in the absence or presence of 10 μM of SB203580. The p38 SAPK dependent genes are highlighted. Click here for file

Additional file 4 **4-fold down-regulated genes upon transient p38 SAPK activation**. List of unique Ensemble-Id genes down-regulated 2.5 fold over a pool of control cells upon a transient p38 SAPK activation. Wild type MEFs and p38α^-/- ^knock out MEFs cells were stimulated with 100 ng/ml TNFα, 25 ng/ml anisomycin and 100 mM NaCl for the indicated times in the absence or presence of 10 μM of SB203580. The p38 SAPK dependent genes are highlighted. Click here for file

Additional file 5 **GO statistic analysis**. Statistics showing significant (p-value ≤ 0.05 and multiple test corrected p-value) in the GO cell component, molecular function and biological processes enriched by the treatments NaCl, anisomycin and TNFα. Total observed genes and total gene number in a given category is also shown. Click here for file

Additional file 6 **GO analysis miniweb**. Interactive miniweb showing the treatment and NaCl time course GO analysis with access to the up-regulated genes found in every category. Files can be open with any standard web browser. Click here for file

Additional file 7 **Supplementary figure 1: The TNFα gene network**. The TNFα gene network inferred by the Ingenuity Pathway software is related to the control of the Immune Response and Immunological Disease. The network genes shaded in grey are up-regulated by the treatment. The network genes in white are not up-regulated by the treatment. Solid arrows indicate direct interactions. Broken arrows indicate indirect interactions. Click here for file

Additional file 8 **Supplementary figure 2: The anisomycin gene network**. The anisomycin gene network inferred by the Ingenuity Pathway software is related to the control of Cell Development and Gene Expression. Click here for file

Additional file 9 **Supplementary figure 3: The NaCl (2 h) gene network**. The NaCl gene network at 2 h inferred by the Ingenuity Pathway software is related to the control of Cell Cycle and Post-translational Modification. Click here for file

Additional file 10 **Supplementary figure 4: The common response gene network**. The common response gene network inferred by the Ingenuity Pathway software is related to the control of Gene Expression and Cancer. Click here for file
