# Supplementary material for: Whole genome analysis of p38 SAPK-mediated gene expression upon stress
Source: BMC Genomics. 2010 Mar 1;11:144. doi: 10.1186/1471-2164-11-144 (PMC2842250; doi:10.1186/1471-2164-11-144)

Whole genome analysis of p38 SAPK-mediated gene expression upon stress

Microarray data diagnosis (Microarray Quality Metrics Analysis)

The quality of the background corrected and normalized microarray data was diagnosed by the Bioconductor package arrayQualityMetrics for the two channels (Cy5 and Cy3).

Quality analysis of microarray data is presented separately for better visualization in the following three groups:

a) Group 1: Treatment and Time Course
b) Group 2: p38α SAPK knock out MEFs
c) Group 3: SB203580 treated wild type MEFs

Every group is divided in two sections:

Section 1: Individual Array Quality; MA plots

$M = \log_2(I_1) - \log_2(I_2)$
$A = 1/2\ (\log_2(I_1)+\log_2(I_2))$
where I_1 and I_2 are the intensities of the two channels. Typically, it is expected that the mass of the distribution in an MA plot to be concentrated along the M = 0 axis, and there should be no trend in the mean of M as a function of A. A trend in the lower range of A usually indicates that the arrays have different background intensities. A trend in the upper range of A usually indicates saturation of the measurements.

Section 2: Array intensity distributions; Box plots and Density plots

Box plots of the $\log_2$(Intensities). Each box corresponds to one array. Array duplicates are shown side by side. The left panel corresponds to the red channel. The middle panel corresponds to the green channel. The right panel shows the box plots of $\log_2$(ratio). Box plots are a graphical representations that summarizes the distribution of probe intensities across all arrays. It comprises the smallest observation, lower quartile, median, upper quartile and largest observation. Typically, it is expected all boxes to have similar size and median.

Density plots show density estimates (smoothed histograms) of the data. Typically, the distributions of the arrays should have similar shapes and ranges. Arrays whose distributions are very different from the others can be considered for possible problems.

a) Group 1: Treatment and Time Course

Array Summary

Array #	Array Name
1	Aniso_Set1
2	Aniso_Set2
3	TNFa_Set1
4	TNFa_Set2
5	NaCl_45_Set1
6	NaCl_45_Set2
7	NaCl_2h_Set1
8	NaCl_2h_Set2
9	NaCl_8h_Set1
10	NaCl_8h_Set2

Section 1: Individual array quality (MA plots)

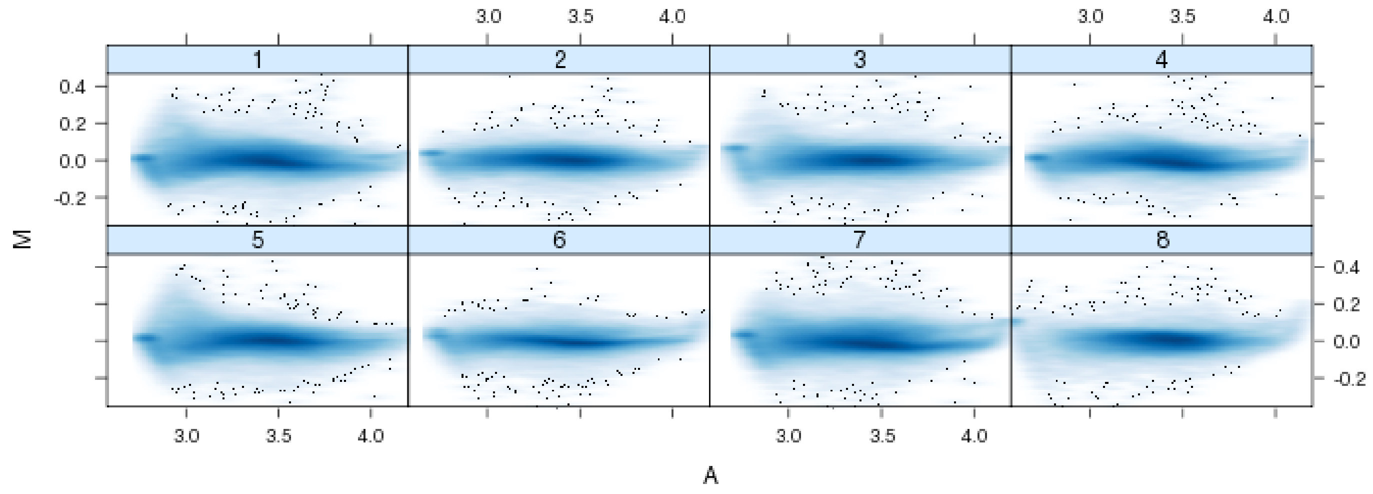

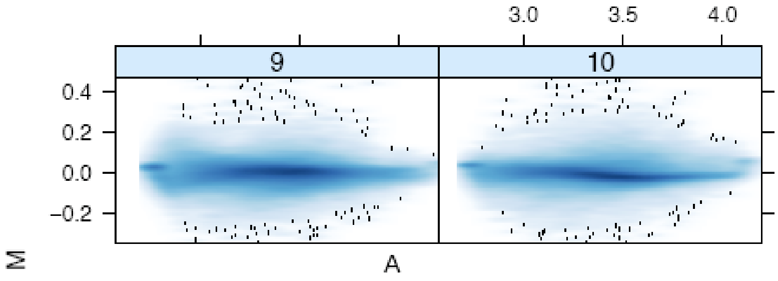

Section 2: **Array intensity distributions; Box plots and Density plots**

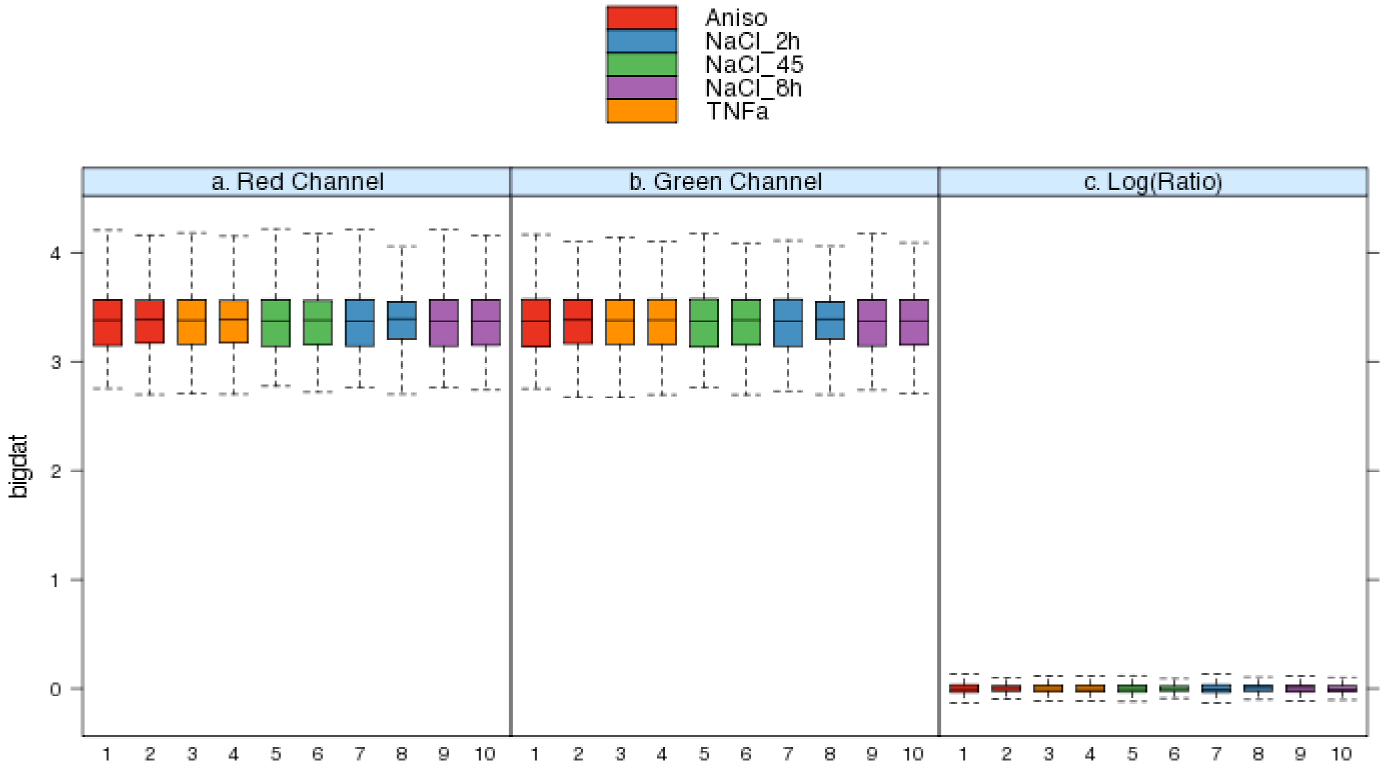

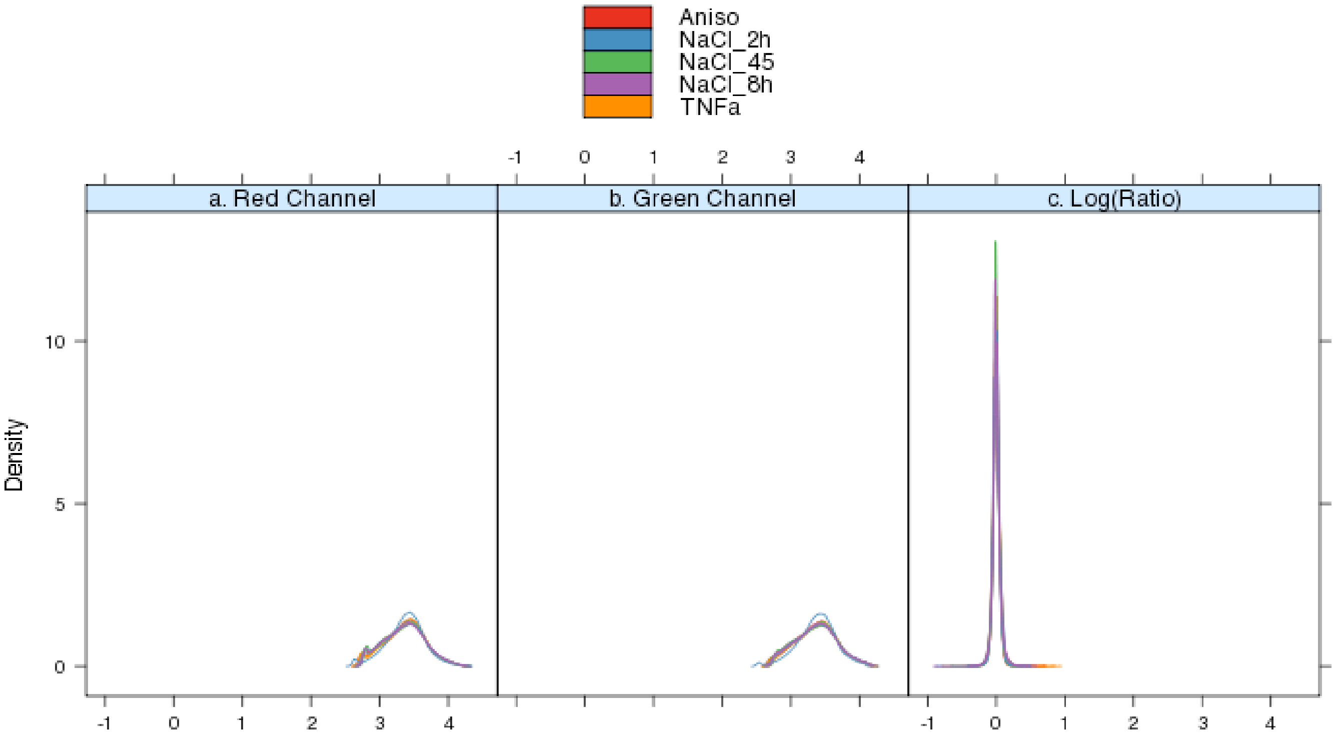

b) Group 2: p38α SAPK knock out MEFs

Array Summary

Array #	Array Name
1	KO_No_Treatment_Set1
2	KO_No_Treatment_Set2
3	KO_Aniso_Set1
4	KO_Aniso_Set2
5	KO_TNFa_Set1
6	KO_TNFa_Set2
7	KO_NaCl_Set1
8	KO_NaCl_Set2

Section 1: Individual array quality; MA plots

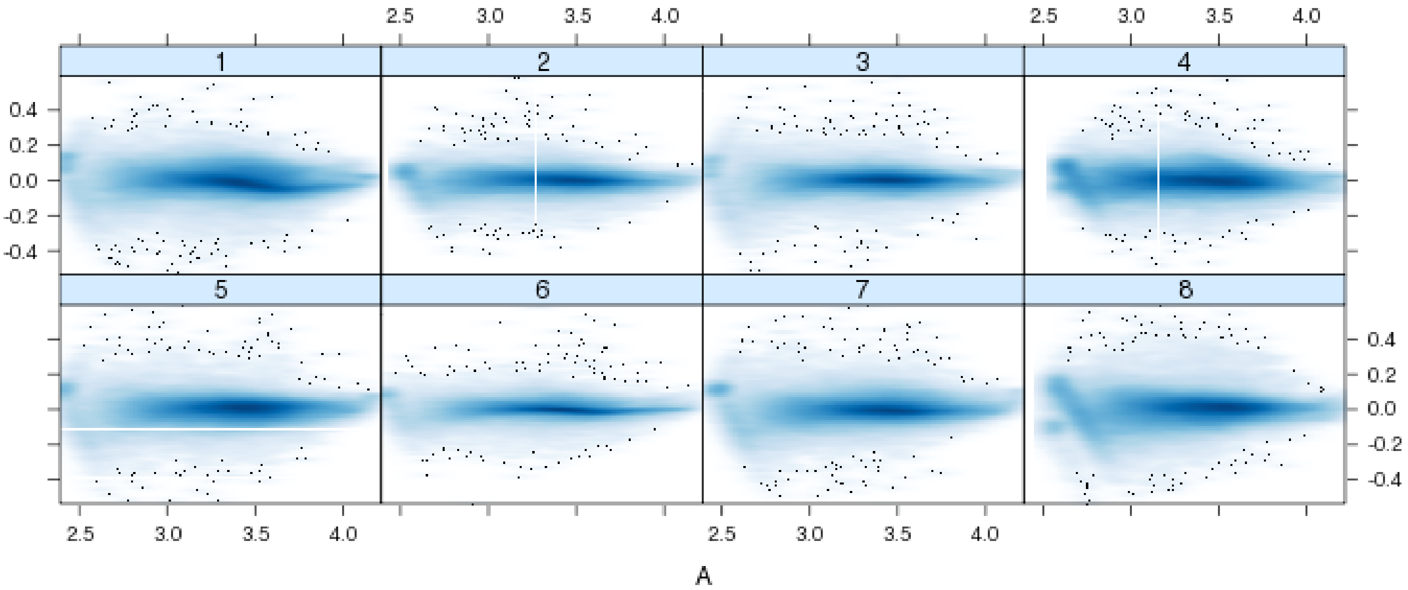

Section 2: Array intensity distributions; Box plots and Density plots

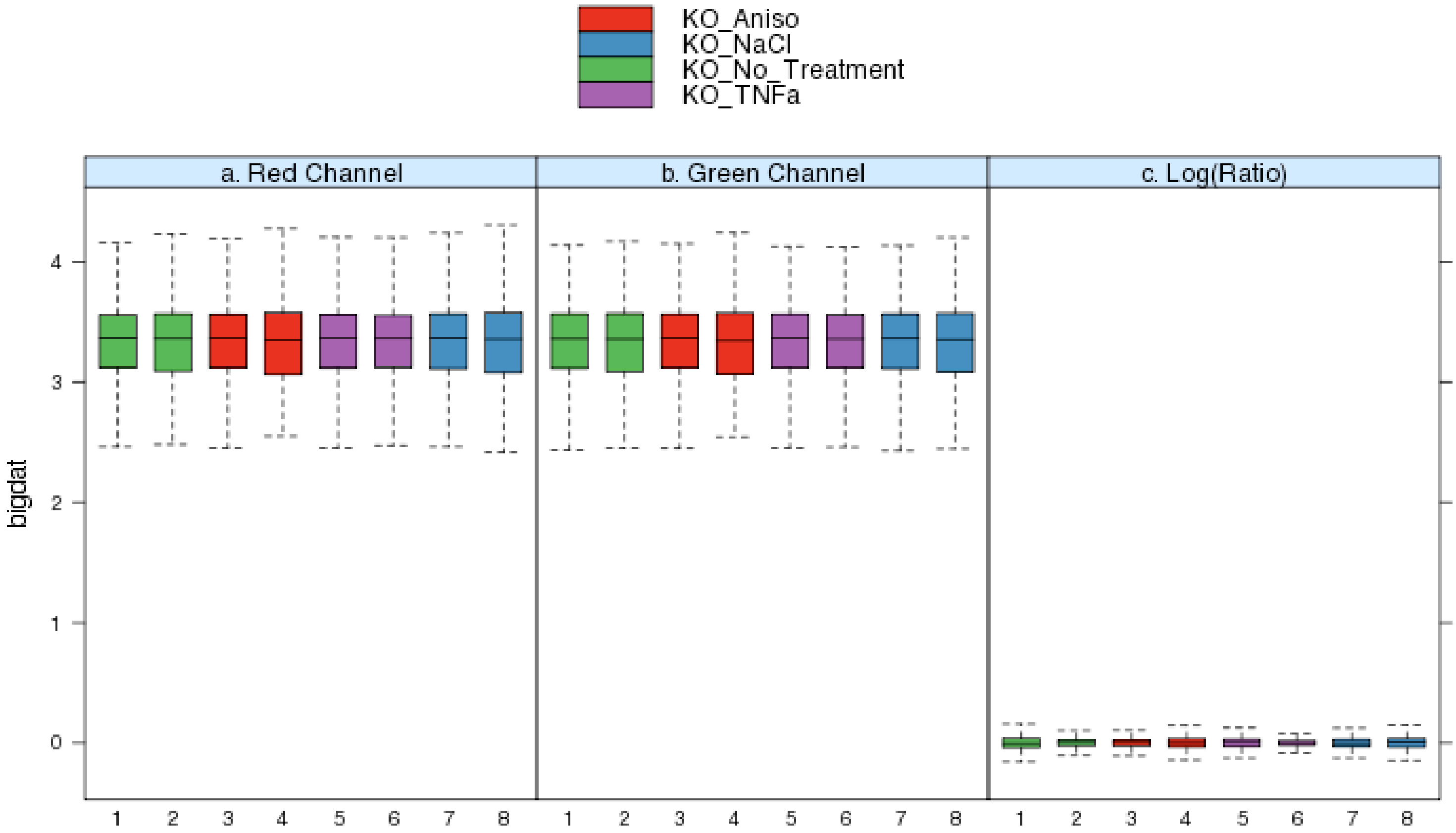

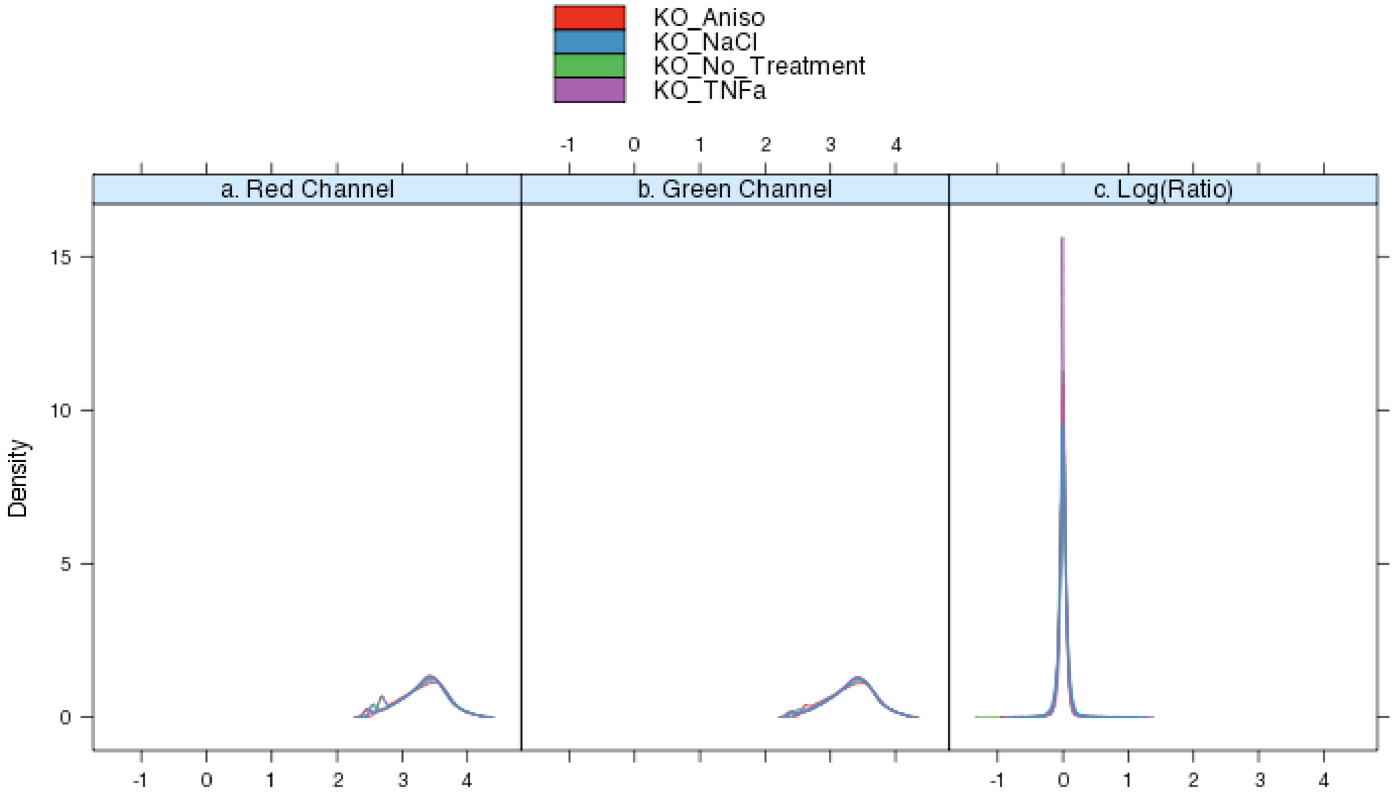

c) Group 3: SB203580 treated wild type MEFs

Array Summary

Array #	Array Name
1	SB_Set1
2	SB_Set2
3	Aniso_SB_Set1
4	Aniso_SB_Set2
5	TNFa_SB_Set1
6	TNFa_SB_Set2
7	NaCl_SB_45_Set1
8	NaCl_SB_45_Set2
9	NaCl_SB_2h_Set1
10	NaCl_SB_2h_Set2
11	NaCl_SB_8h_Set1
12	NaCl_SB_8h_Set2

Section 1: Individual array quality; MA plots

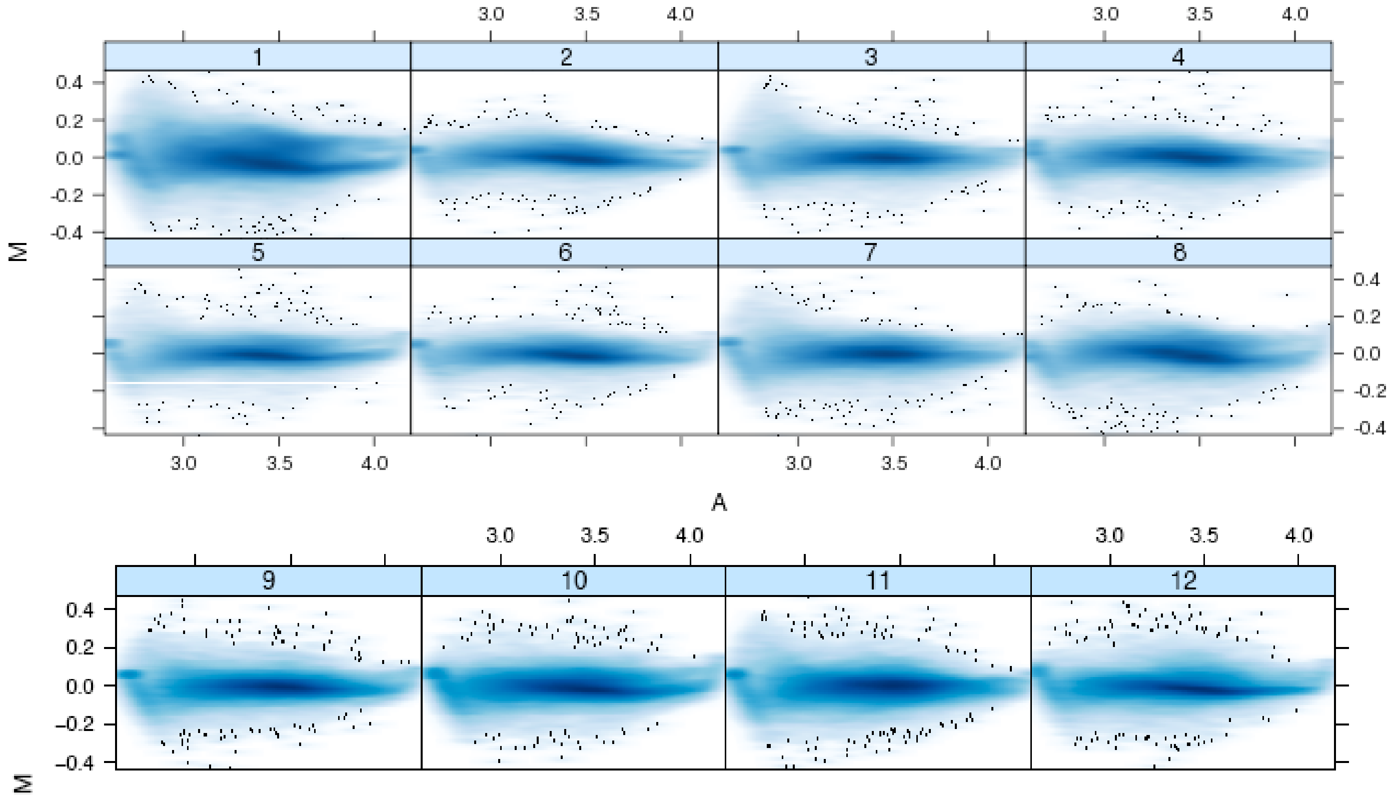

Section 2: **Array intensity distributions; Box plots and Density plots**

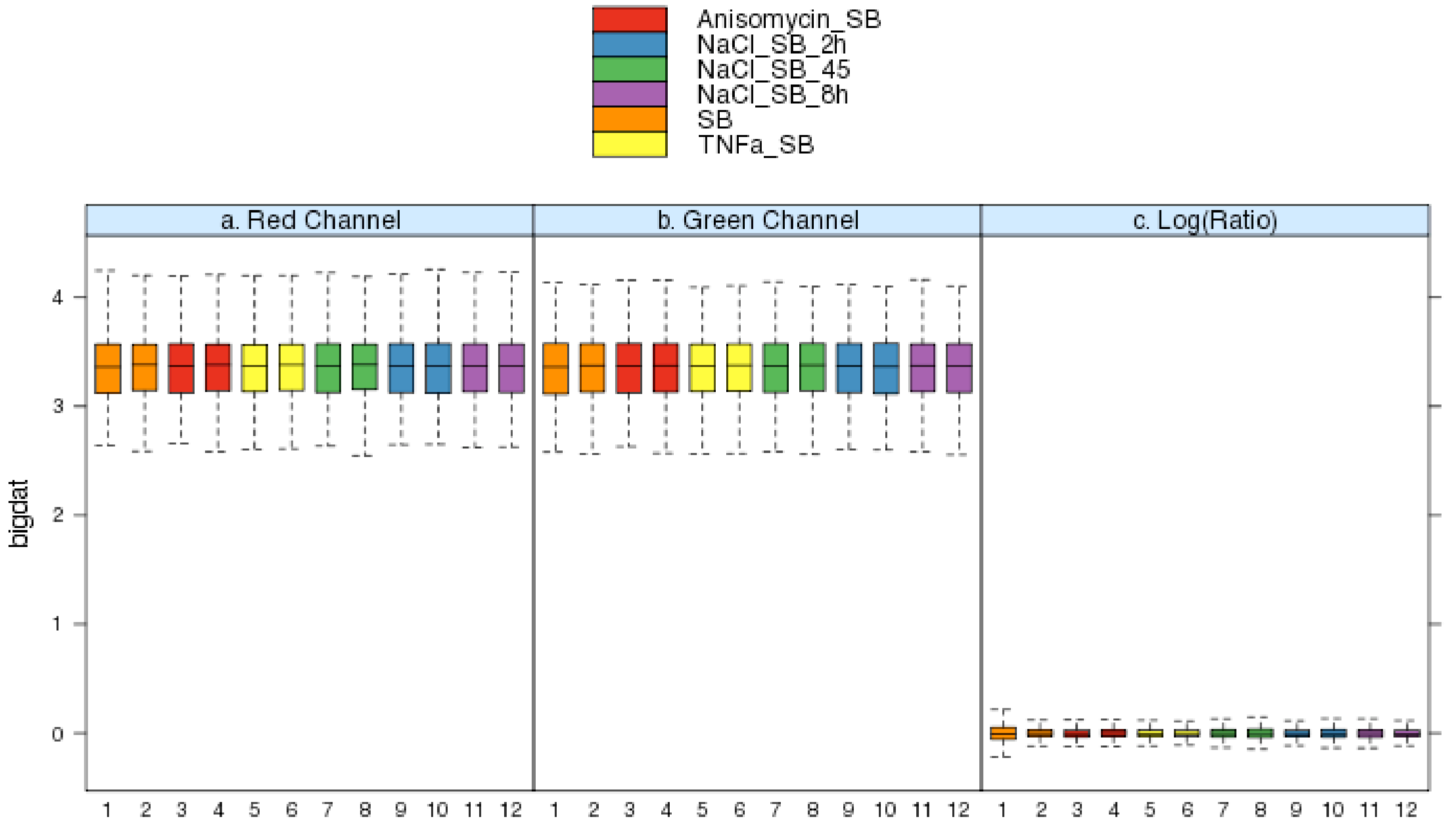

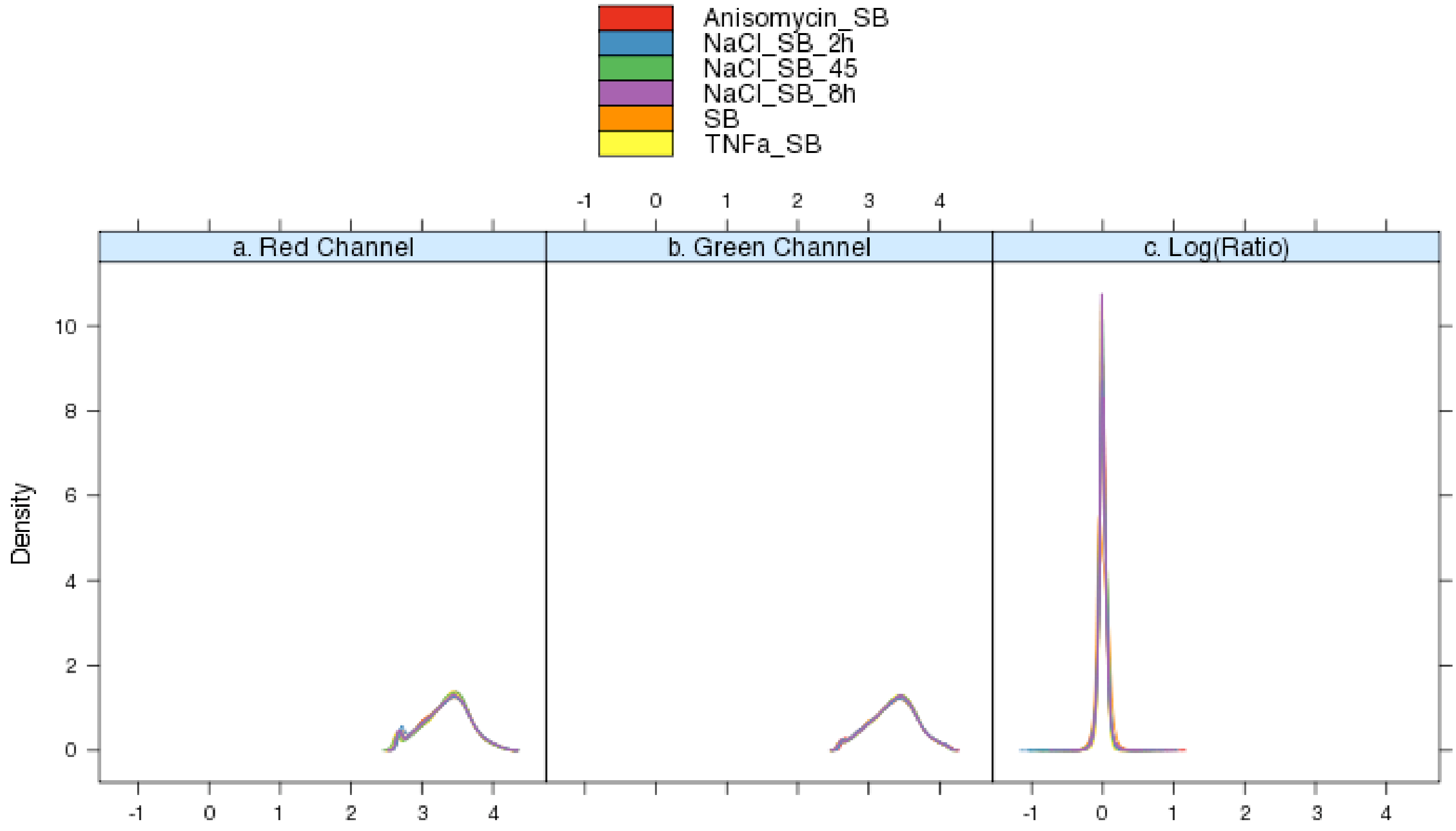

Supplement: Additional file 2 — Microarray Quality Metrics Analysis. For a better visualization the microarray data the diagnosis has been split in three groups named 1; Treatment and Time Course. 2; p38α SAPK knock out MEFs and 3; SB203580 treated wildt type MEFs. Section 1 analyses the individual array quality through MA. The mass distribution in an MA plot is expected to be concentrated along the M = 0 axis and there should be no trend in the mean of M as a function of A. A trend in the lower range of A usually indicates that the arrays have different background intensities. A trend in the upper range of A usually indicates saturation of the measurements. Section 2 analyses the array intensity distributions through Box plots and Density plots. Each Box box corresponds to one array. Array duplicates are shown side by side. The left panel corresponds to the red Cy5 channel. The middle panel corresponds to the green Cy3 channel. The right panel shows the Box plots of the log2 Cy5/Cy3 ratio. Box plots are a graphical representation that summarises the distribution of probe intensities across all arrays. It comprises the smallest observation, lower quartile, median, upper quartile and largest observation. All boxes are expected to have similar size and median. Density plots show smoothed histograms of the array signal intensities. The left panel corresponds to the red Cy5 channel. The middle panel corresponds to the green Cy3 channel. The right panel shows the Box plots of the log2 Cy5/Cy3 ratio. The distribution of the arrays is expected to have similar shapes and ranges. [file 1471-2164-11-144-S2.PDF]
