# Supplementary material for: Whole genome analysis of p38 SAPK-mediated gene expression upon stress
Source: BMC Genomics. 2010 Mar 1;11:144. doi: 10.1186/1471-2164-11-144 (PMC2842250; doi:10.1186/1471-2164-11-144)
Supplement: Additional file 7 — Supplementary figure 1: The TNFα gene network. The TNFα gene network inferred by the Ingenuity Pathway software is related to the control of the Immune Response and Immunological Disease. The network genes shaded in grey are up-regulated by the treatment. The network genes in white are not up-regulated by the treatment. Solid arrows indicate direct interactions. Broken arrows indicate indirect interactions. [file 1471-2164-11-144-S7.PDF]

Figure S1. TNFα gene Network. Immune Response. Immunological Disease. Connective Tissue Disorder

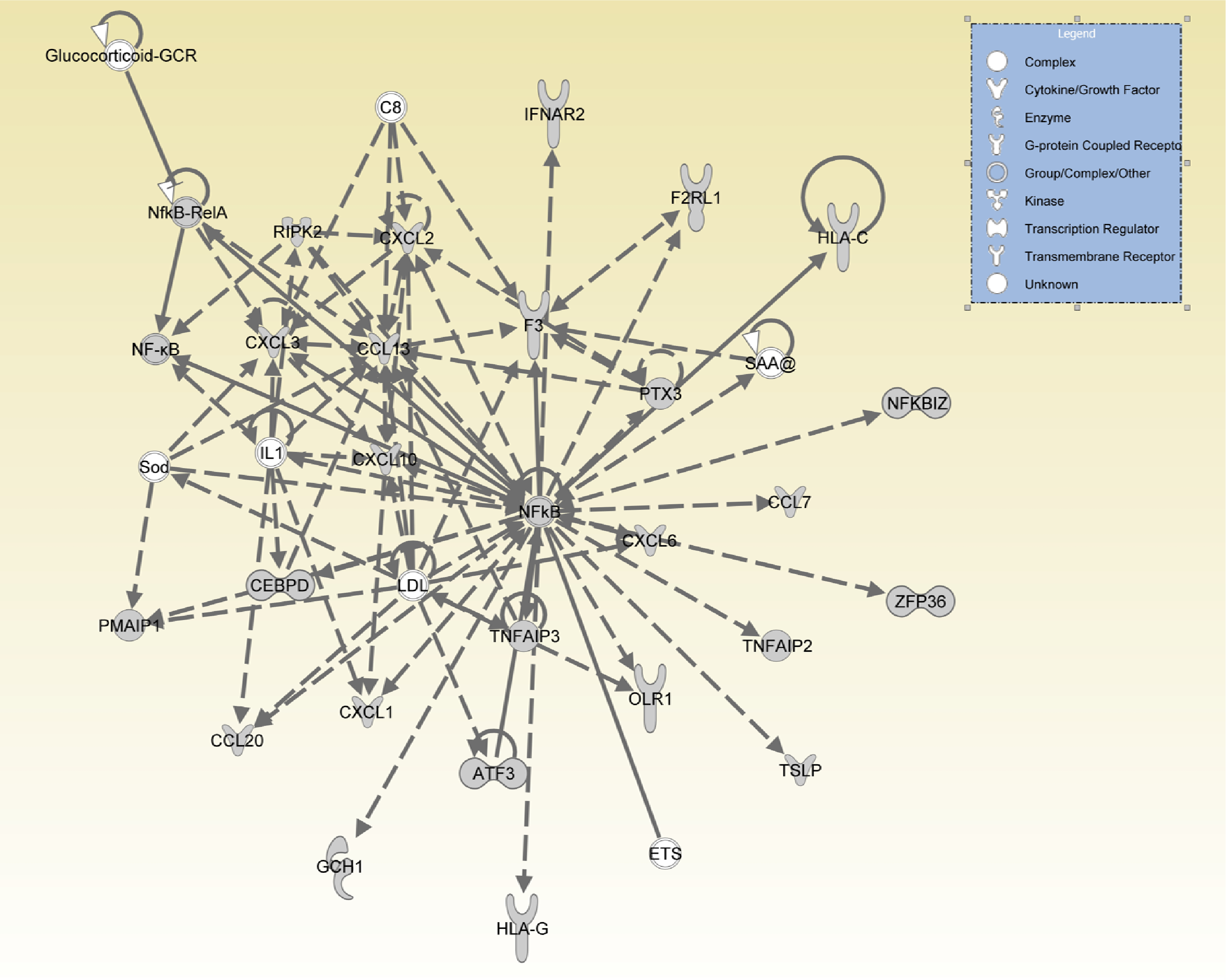

© 2000-2009 Ingenuity Systems, Inc. All rights reserved.
