# Supplementary figures and images for: Whole genome analysis of p38 SAPK-mediated gene expression upon stress
Source: BMC Genomics. 2010 Mar 1;11:144. doi: 10.1186/1471-2164-11-144 (PMC2842250; doi:10.1186/1471-2164-11-144)

**Figure S2. Anisomycin gene Network. Gene Expression and Development**

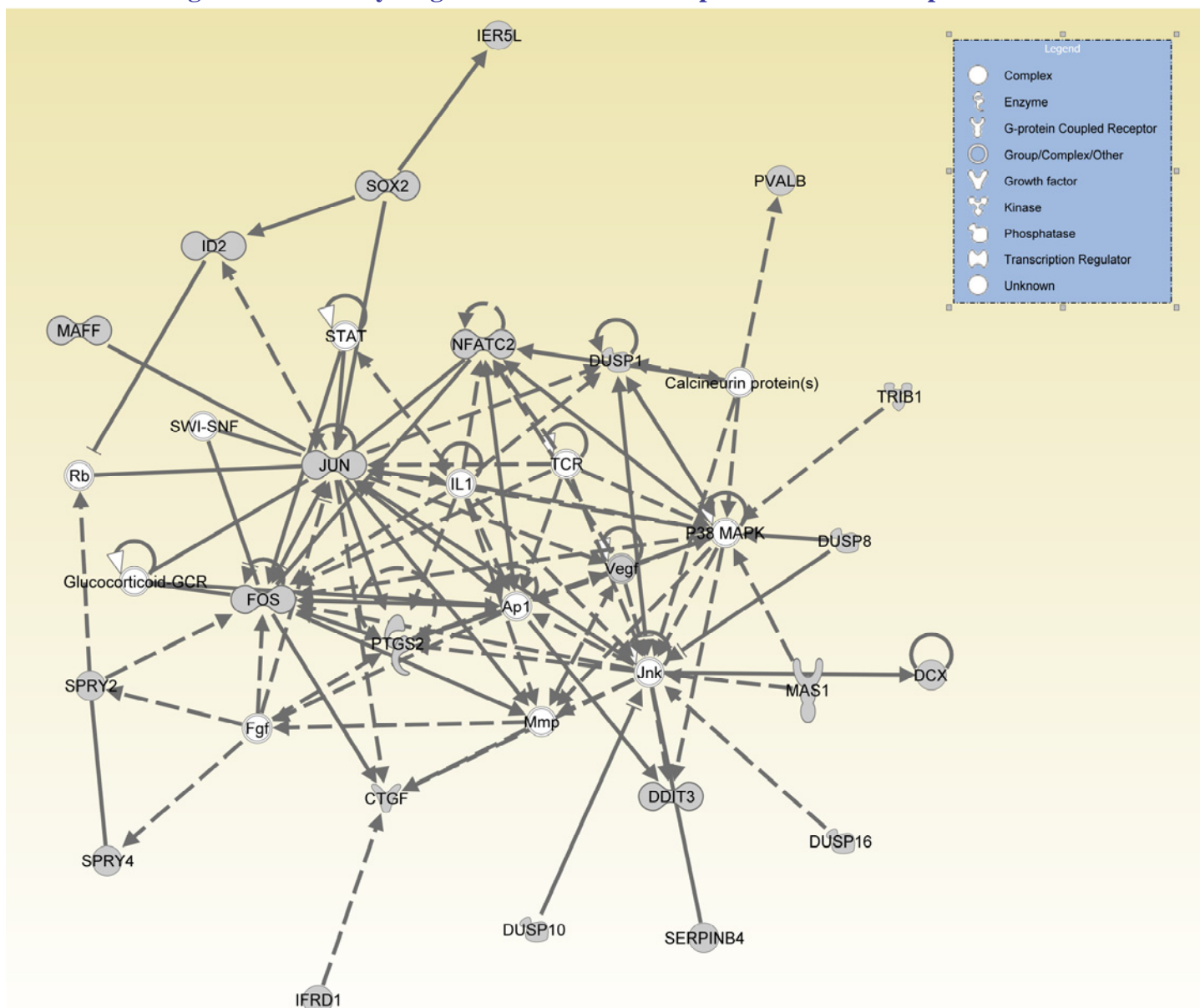

Supplement: Additional file 8 — Supplementary figure 2: The anisomycin gene network. The anisomycin gene network inferred by the Ingenuity Pathway software is related to the control of Cell Development and Gene Expression. [file 1471-2164-11-144-S8.PDF]

**Figure S4. Common gene Network. Gene Expression and Cancer**

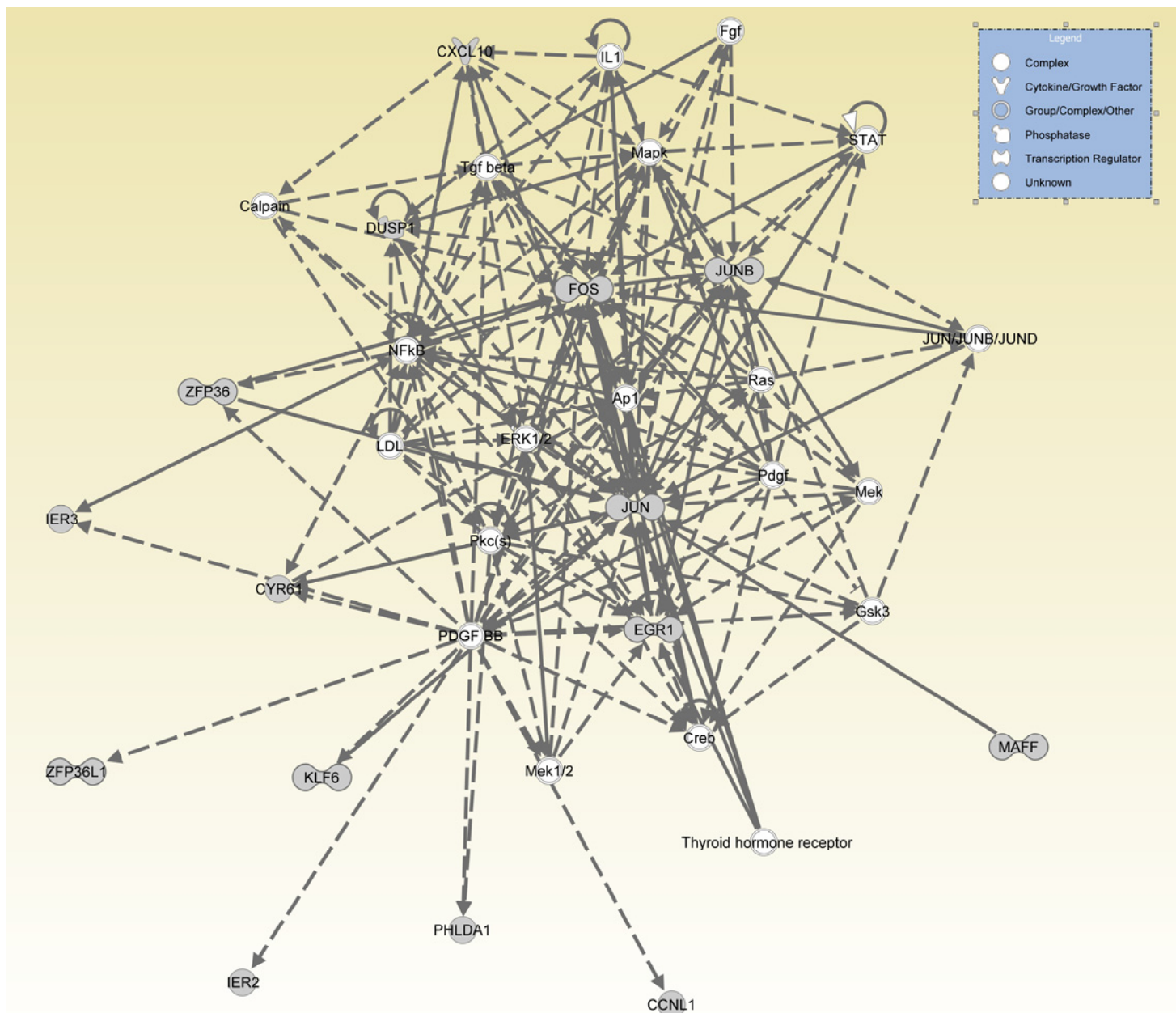

Supplement: Additional file 10 — Supplementary figure 4: The common response gene network. The common response gene network inferred by the Ingenuity Pathway software is related to the control of Gene Expression and Cancer. [file 1471-2164-11-144-S10.PDF]
