# Supplementary material for: Whole genome analysis of p38 SAPK-mediated gene expression upon stress
Source: BMC Genomics. 2010 Mar 1;11:144. doi: 10.1186/1471-2164-11-144 (PMC2842250; doi:10.1186/1471-2164-11-144)
Supplement: Additional file 9 — Supplementary figure 3: The NaCl (2 h) gene network. The NaCl gene network at 2 h inferred by the Ingenuity Pathway software is related to the control of Cell Cycle and Post-translational Modification. [file 1471-2164-11-144-S9.PDF]

Figure S3. NaCl (2h) gene Network. Cell Cycle. Amino Acid Metabolism. Post-Translational Modification

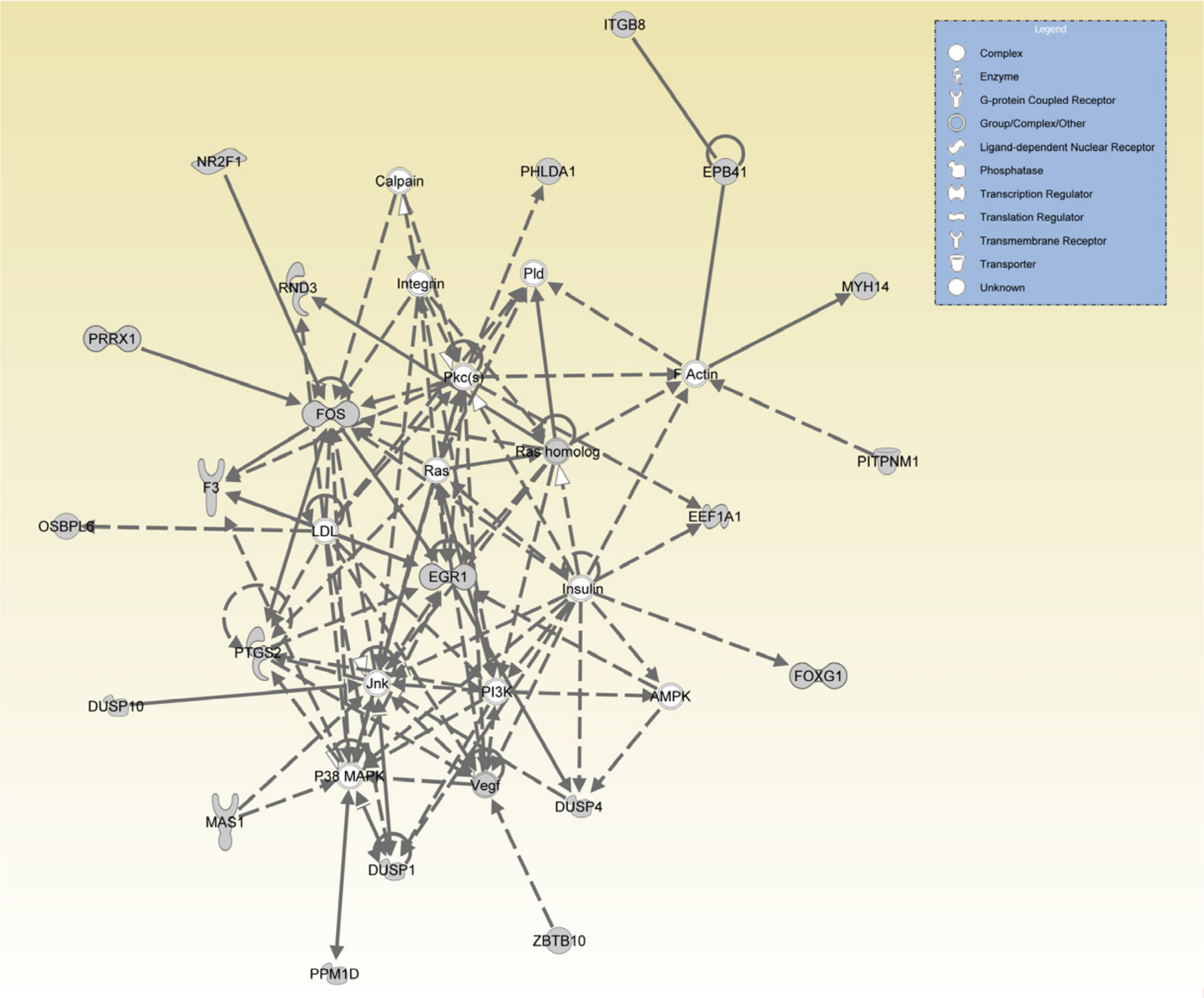

© 2000-2009 Ingenuity Systems, Inc. All rights reserved.
